# Supplementary material for: Diagnostic accuracy of computed tomography in adults with suspected acute appendicitis at the emergency department in a private tertiary hospital in Tanzania
Source: PLoS One. 2022 Oct 27;17(10):e0276720. doi: 10.1371/journal.pone.0276720 (PMC9612457; doi:10.1371/journal.pone.0276720)
Supplement: Attachment — Submitted filename: PONE-D-21-13993_R3_reviewer.pdf [file pone.0276720.s005.pdf]

PLOS ONE

DIAGNOSTIC ACCURACY OF COMPUTED TOMOGRAPHY IN ADULTS WITH SUSPECTED ACUTE APPENDICITIS AT THE EMERGENCY DEPARTMENT IN A PRIVATE TERTIARY HOSPITAL IN TANZANIA

--Manuscript Draft--

**Manuscript Number:**	PONE-D-21-13993R3
**Article Type:**	Research Article
**Full Title:**	DIAGNOSTIC ACCURACY OF COMPUTED TOMOGRAPHY IN ADULTS WITH SUSPECTED ACUTE APPENDICITIS AT THE EMERGENCY DEPARTMENT IN A PRIVATE TERTIARY HOSPITAL IN TANZANIA
**Short Title:**	DIAGNOSTIC ACCURACY OF CT IN ADULTS WITH SUSPECTED ACUTE APPENDICITIS AT THE EMD IN IN TANZANIA
**Corresponding Author:**	Masawa Klint Nyamuryekung'e, MD., MMED.<br>Aga Khan Hospital Dar es Salaam<br>Dar es Salaam, TANZANIA, UNITED REPUBLIC OF
**Keywords:**	Acute appendicitis; CT abdomen; sensitivity; Diagnostic Accuracy; Africa
**Abstract:**	Introduction<br>The increasing incidence of acute appendicitis in sub-Saharan Africa emphasizes the need for accurate and reliable diagnostic tools. However, the variability in the diagnostic performance of computed tomography for suspected acute appendicitis coupled with comparatively higher negative appendectomy rates in this setting highlight a possible concern regarding the diagnostic accuracy.<br>This study evaluated the diagnostic accuracy of a computed tomography scan for suspected acute appendicitis at the emergency department in Tanzania.<br>Methods<br>A retrospective diagnostic accuracy study was conducted from July to October 2020. All patients above 14 years of age who presented at the emergency department with right iliac fossa abdominal pain of fewer than ten days and underwent computed tomography for suspected acute appendicitis were evaluated, and the Alvarado score was computed. Histological diagnosis and clinical follow-up of 14 days were considered the reference standard. Ethical clearance was sought from the Aga Khan University Ethical review committee.<br>Results<br>176 patients were included in this study. The sensitivity, specificity, and diagnostic accuracy were 100% (95% CI 91.8-100), 96.9% (95% CI 92.2-99.1), and 96.9% (95% CI 93.1-98.3), respectively. The mean Alvarado score in those without acute appendicitis was 4 (95% C.I 3.7-4.3) compared to a mean score of 6.6 (95% CI 6.0-7.2) amongst those with acute appendicitis. The area under the receiver operator characteristics curve of computed tomography was 0.984, and that of the Alvarado score was 0.841.<br>Conclusions<br>The diagnostic performance of computed tomography in this study is similar to that established elsewhere. However, the AS is not routinely used for the initial screening of suspected acute appendicitis patients. A threshold of AS of 4 as a guide to conduct CT for suspected AA would have decreased CT use by 50%, and missed 4 cases. Implementation studies that address AS use should be conducted.
**Order of Authors:**	Masawa Klint Nyamuryekung'e, MD., MMED.
	Miten Ramesh Patel
	Ahmed Jusabani
	Aliakber Zehri
	Athar Ali
**Response to Reviewers:**	Comments to the Author<br><br>Reviewer #1:

Powered by Editorial Manager® and ProduXion Manager® from Aries Systems Corporation

	The authors submitted a revised manuscript and responded to the questions. The authors stated that “The Alvarado score (AS) was not documented. However, based on symptoms assessed and the results of the CBC, the AS was computed for each patient. The mean AS was 4.64 (95% CI 4.32-4.97), those with AS of 1 to 4 were 50.6%”. As the authors’ replied, the inclusion criteria of the study were too loose. The AS of the patients less than 4 were 50.6% of the study patients, who should not receive abdominal CT for suspect acute appendicitis. The loose criteria made the high percentage (39%) of urolithiasis in the study population. The conclusion: “The commonest alternative diagnosis was urolithiasis and should be considered during the evaluation of adult patients with suspected acute appendicitis.” is inappropriate. Emergency physicians usually perform more examinations, such as urinalysis and abdominal ultrasonography for patients with right lower abdominal colic pain before CT. The diagnosis of urolithiasis is easy to identify by these examinations. If we adopt the authors’ criteria, we would perform CT for every patient with right lower abdominal colic pain.<br><br>Response:<br>Thank you for your comments. For uniformity, the inclusion criteria were adopted from a meta-analysis and systematic on the same subject by Bo Rud et al, 2019. The use of USS prior to CT introduces a confounding effect on the assessment of CT’s diagnostic performance, and this is an exclusion criterion, in this study and the systematic review quoted above.<br>The concerns on the use of AS have been analyzed and discussed, using the AS threshold of 4 would have resulted in 50% less CT scans, and missed 4 AA.<br>Recommendations for the use of AS in assessing patients with suspected acute appendicitis have been emphasized.<br>The recommendation by the authors referred to above had been rectified in an earlier revised version and is not present in the current revised version. Results of urinalysis done have been described and analyzed.<br><br>Reviewer #2:<br>This is my second review of this manuscript. The authors have replied and made revisions regarding previous reviewers’ major concerns. Yet there remained more issues.<br>1. In my first comment, the authors stated that “The image interpreters were four consultant medical radiologists rotating through an on-call schedule. Please give the Kappa value between these four radiologists”. And the authors replied as “Each radiologist interpreted the results independently, depending on when he/she was on call. This has been clarified in the manuscript”. For this comment, my question was “whether the same image was interpreted by two or more radiologists or just one? If two or more radiologists have interpreted the same image, I would like to know how the degree of the consistent outcome through giving the kappa values? But if just one radiologist interpreted for one image, this may be lead to an inconsistent outcome and must have been addressed.<br>2. In my first comment, I have stated that “Please give the 95% confidence intervals for sensitivity, specificity, diagnostic accuracy, positive and negative predictive value”, but I suggest the authors revise the terms of confidence intervals into more formula terms. For example, (95% Confidence Interval of 0.79 to 0.96) to (95% C.I.: 0.79-0.96). the same terms through this manuscript like this term also please address accordingly. Additionally, do not use the percentage and decimal point interchangeably, please unify.<br>Response to comment 1.<br>The concern for four independent radiologists evaluating the CT introduces inter-observer variability. This is has been discussed as a limitation of this study.<br>Response to comment 2.<br>This has been rectified. Thank you for your comments.
**Additional Information:**	
**Question**	**Response**
**Financial Disclosure**<br><br>Enter a financial disclosure statement that describes the sources of funding for the	The authors received no funding for this work

work included in this submission. Review the submission guidelines for detailed requirements. View published research articles from *PLOS ONE* for specific examples.<br><br>This statement is required for submission and **will appear in the published article** if the submission is accepted. Please make sure it is accurate.<br><br>**Unfunded studies**<br>Enter: *The author(s) received no specific funding for this work.*<br><br>**Funded studies**<br>Enter a statement with the following details:<br>• Initials of the authors who received each award<br>• Grant numbers awarded to each author<br>• The full name of each funder<br>• URL of each funder website<br>• Did the sponsors or funders play any role in the study design, data collection and analysis, decision to publish, or preparation of the manuscript?<br>• **NO** - Include this sentence at the end of your statement: *The funders had no role in study design, data collection and analysis, decision to publish, or preparation of the manuscript.*<br>• **YES** - Specify the role(s) played.<br><br>* typeset	
**Competing Interests**<br><br>Use the instructions below to enter a competing interest statement for this submission. On behalf of all authors, disclose any competing interests that could be perceived to bias this work—acknowledging all financial support and any other relevant financial or non-financial competing interests.<br><br>This statement is required for submission and **will appear in the published article** if the submission is accepted. Please make sure it is accurate and that any funding sources listed in your Funding Information	The authors have declared that no competing interests exist

later in the submission form are also declared in your Financial Disclosure statement.<br><br>View published research articles from *PLOS ONE* for specific examples.<br><br>**NO authors have competing interests**<br><br>Enter: *The authors have declared that no competing interests exist.*<br><br>**Authors with competing interests**<br><br>Enter competing interest details beginning with this statement:<br><br>*I have read the journal's policy and the authors of this manuscript have the following competing interests: [insert competing interests here]*<br><br>* typeset	
**Ethics Statement**<br><br>Enter an ethics statement for this submission. This statement is required if the study involved:<br><br>• Human participants<br>• Human specimens or tissue<br>• Vertebrate animals or cephalopods<br>• Vertebrate embryos or tissues<br>• Field research<br><br>Write "N/A" if the submission does not require an ethics statement.<br><br>General guidance is provided below. Consult the submission guidelines for detailed instructions. **Make sure that all information entered here is included in the Methods section of the manuscript.**	Ethical clearance for the study protocol was sought from the Aga Khan University Ethical review committee, AKU-ERC, EA, with a reference number of AKU/2020/0170/fb. Consent was waived as this was a retrospective chart review and the data collected was de identified.

**Format for specific study types**<br><br>**Human Subject Research (involving human participants and/or tissue)**<br>• Give the name of the institutional review board or ethics committee that approved the study<br>• Include the approval number and/or a statement indicating approval of this research<br>• Indicate the form of consent obtained (written/oral) or the reason that consent was not obtained (e.g. the data were analyzed anonymously)<br><br>**Animal Research (involving vertebrate animals, embryos or tissues)**<br>• Provide the name of the Institutional Animal Care and Use Committee (IACUC) or other relevant ethics board that reviewed the study protocol, and indicate whether they approved this research or granted a formal waiver of ethical approval<br>• Include an approval number if one was obtained<br>• If the study involved *non-human primates*, add *additional details* about animal welfare and steps taken to ameliorate suffering<br>• If anesthesia, euthanasia, or any kind of animal sacrifice is part of the study, include briefly which substances and/or methods were applied<br><br>**Field Research**<br><br>Include the following details if this study involves the collection of plant, animal, or other materials from a natural setting:<br>• Field permit number<br>• Name of the institution or relevant body that granted permission	
**Data Availability**<br><br>Authors are required to make all data underlying the findings described fully available, without restriction, and from the time of publication. PLOS allows rare exceptions to address legal and ethical concerns. See the PLOS Data Policy and FAQ for detailed information.	No - some restrictions will apply

Powered by Editorial Manager® and ProduXion Manager® from Aries Systems Corporation

A Data Availability Statement describing where the data can be found is required at submission. Your answers to this question constitute the Data Availability Statement and **will be published in the article**, if accepted.<br><br>**Important:** Stating 'data available on request from the author' is not sufficient. If your data are only available upon request, select 'No' for the first question and explain your exceptional situation in the text box.<br><br>Do the authors confirm that all data underlying the findings described in their manuscript are fully available without restriction?	
**Describe where the data may be found in full sentences. If you are copying our sample text, replace any instances of XXX with the appropriate details.**<br><br>• If the data are **held or will be held in a public repository**, include URLs, accession numbers or DOIs. If this information will only be available after acceptance, indicate this by ticking the box below. For example: *All XXX files are available from the XXX database (accession number(s) XXX, XXX.*).<br>• If the data are all contained **within the manuscript and/or Supporting Information files**, enter the following: *All relevant data are within the manuscript and its Supporting Information files.*<br>• If neither of these applies but you are able to provide **details of access elsewhere**, with or without limitations, please do so. For example:<br><br>*Data cannot be shared publicly because of [XXX]. Data are available from the XXX Institutional Data Access / Ethics Committee (contact via XXX) for researchers who meet the criteria for access to confidential data.*<br><br>*The data underlying the results presented in the study are available from (include the name of the third party*	The data in the study is the property of AKU, Dar Campus (third party); The data points can only be available on request to Agha Khan University, The Research Administrative office, Ms. Mwanaarab R. Sibuma, The Aga Khan University Medical College, P.O. Box 38129, Dar es salaam Tanzania, Cell: +255 682 00097, e-mail: mwanaarab.sibuma@aku.edu

Powered by Editorial Manager® and ProduXion Manager® from Aries Systems Corporation

and contact information or URL).<br>• This text is appropriate if the data are owned by a third party and authors do not have permission to share the data.<br><br>* typeset	
Additional data availability information:	

Powered by Editorial Manager® and ProduXion Manager® from Aries Systems Corporation

Manuscript Click here to access/download;Manuscript;Manuscript.docx

Diagnostic accuracy of computed tomography in adults with suspected acute appendicitis at the emergency department in a private tertiary hospital in Tanzania

Masawa K. Nyamuryekung'e[1*], Miten R. Patel[1¶], Ahmed Jusabani[1¶], Ali A. Zehri[1, #a, ¶], Athar Ali[1¶]

[1]Department of Surgery, The Aga Khan Hospital, Dar es salaam, Tanzania

[#a]Current Address: Department of Surgery, Mukhtar A. Sheikh Hospital, Punjab, Pakistan

*Corresponding Author

E-mail: masawa4@hotmail.com (MKN)

[¶] These authors contributed equally to this work

Acknowledgments: We thank Kasusu Klint Nyamuryekung'e (DDS, MPhil Int. Health, PhD) for his assistance and inputs in the statistical analysis and preparation of the manuscript.

ABSTRACT

Introduction

The increasing incidence of acute appendicitis in sub-Saharan Africa emphasizes the need for accurate and reliable diagnostic tools. However, the variability in the diagnostic performance of computed tomography for suspected acute appendicitis coupled with comparatively higher negative appendectomy rates in this setting highlight a possible concern regarding the diagnostic accuracy.

This study evaluated the diagnostic accuracy of a computed tomography scan for suspected acute appendicitis at the emergency department in Tanzania.

Methods

A retrospective diagnostic accuracy study was conducted from July to October 2020. All patients above 14 years of age who presented at the emergency department with right iliac fossa abdominal pain of fewer than ten days and underwent computed tomography for suspected acute appendicitis were evaluated, and the Alvarado score was computed. Histological diagnosis and clinical follow-up of 14 days were considered the reference standard. Ethical clearance was sought from the Aga Khan University Ethical review committee.

Results

176 patients were included in this study. The sensitivity, specificity, and diagnostic accuracy were 100% (95% CI 91.8-100), 96.9% (95% CI 92.2-99.1), and 96.9% (95% CI 93.1-98.3), respectively. The mean Alvarado score in those without acute appendicitis was 4 (95% CI 3.7-4.3) compared to a mean score of 6.6 (95% CI 6.0-7.2) amongst those with acute appendicitis. The area under the

receiver operator characteristics curve of computed tomography was 98.4%, and that of the Alvarado score was 84.1%.

Conclusions

The diagnostic performance of computed tomography in this study is similar to that established elsewhere. However, the Alvarado score is not routinely used for the initial screening of suspected acute appendicitis patients. A threshold of Alvarado score of 4 as a guide to conduct computed tomography for suspected acute appendicitis would have decreased computed tomography use by 50%, and missed 4 cases. Implementation studies that address Alvarado score use should be conducted.

List of Abbreviations

AA- Acute Appendicitis

AS- Alvarado Score

AUROC- Area Under the Receiver Operator Characteristics curve

CT- Computed Tomography

CI- Confidence interval

EMD- Emergency Medicine Department

LR+- Positive Likelihood ratio

LR- Negative Likelihood ratio

ROC- Receiver operator characteristics

SSA- Sub Saharan Africa

WSES- World society of emergency surgery

KEYWORDS: Acute appendicitis, CT abdomen, Sensitivity, Diagnostic accuracy, Africa

DIAGNOSTIC ACCURACY OF COMPUTED TOMOGRAPHY IN ADULTS WITH SUSPECTED ACUTE APPENDICITIS AT THE EMERGENCY DEPARTMENT IN PRIVATE TERTIARY HOSPITAL IN TANZANIA

INTRODUCTION

Acute appendicitis (AA) is the commonest cause of acute abdomen in adults that frequently requires emergency surgical intervention (1). AA has been viewed primarily as a western disease; however, current disease trends have suggested increasing incidence in sub-Saharan Africa (SSA), underscoring its growing significance (2).

An accurate diagnosis of AA can be made clinically about 50% of the time. In the remaining instances, diagnosis solely through clinical findings may be challenging (3). There is an increased likelihood of diagnosis delays or misdiagnosis in this scenario. This may result in higher appendicular perforation and negative appendectomy rates (4, 5). To improve the diagnostic accuracy of AA, medical imaging tools are frequently utilized in addition to clinical findings. The abdominal computed tomography (CT) is the commonest preferred medical imaging used for this purpose in nonpregnant adults (6). It allows for visualization of the appendix, evaluation of features known to predict AA and determining alternative pathologies (7, 8).

Multiple systematic reviews and meta-analyses have established that CT's diagnostic accuracy for suspected AA in nonpregnant adult patients is over 92% (7-12). However, in the pooled meta-analysis studies, the range of sensitivity and specificity of CT’s diagnosis for suspected AA was

72 to 100% and 50 to 100%, respectively (12). Variability of diagnostic accuracy may be due to radiologists' experience, contrast use, differing CT generations, imaging protocols, and acute appendicitis prevalence in the study settings (7, 9, 11, 12).

There have been over ten meta-analyses and systematic reviews conducted to date assessing the diagnostic performance of CT scans for suspected AA in adults, and none include findings from SSA, possibly due to the lack of eligible studies from this area (7-15). This partly reflects the limited resources of health facilities in terms of equipment available, expertise, experience, and possibly the preference of the imaging modality for suspected AA (16-19). The outcomes of performed appendectomies in SSA reveal a negative appendectomy rate of about 17%, compared to 1- 5% in other areas, raising concern for diagnostic accuracy of suspected AA in this region (20, 21).

The current study's main objectives were to determine the sensitivity, specificity, diagnostic accuracy, and predictive values of CT for patients with suspected AA presenting to the emergency medicine department (EMD) of Aga Khan Hospital, Tanzania.

METHODS

Ethical clearance for the study protocol was sought from the Aga Khan University Ethical review committee, AKU-ERC, EA, with a reference number of AKU/2020/0170/fb. Consent was waived as this was a retrospective chart review and the data collected was de identified. The study was conducted from July to October 2020 at a private tertiary teaching hospital in Dar es Salaam, Tanzania. Records of patients aged 14 years and above who presented at the EMD with right iliac fossa colicky abdominal pain for less than 10 days and underwent CT abdomen for suspected AA were included in this study. Pregnant women and those who underwent abdominal-pelvic

ultrasonography prior to the abdominal CT scan were excluded. Sample size estimation based on the Buderer formula of diagnostic accuracy for unknown disease prevalence was employed (22). The assumptions applied were an acceptable marginal error of 0.05, hypothesized sensitivity of 0.95, specificity of 0.94 and prevalence of 0.43, based on Bo Rud, 2019 et al. (12). The required sample size for sensitivity and specificity were 170 and 153 participants, respectively. Consecutive sampling was utilized to include patient records into the study until the sample size was reached.

The index test for suspected AA was a helical CT scan manufactured by Philips, Ingenuity model, 128 slices with 64 detector rows. One of four consultant medical radiologists interpreted the images individually during their on-call schedule. All radiologists had at least trained as Master of Medicine in medical radiology. The target condition was AA, encompassing both simple and complicated presentations. All cases diagnosed by CT to have simple or complicated AA were considered test positive. Alternately, all cases with a normal appendix on CT evaluation or alternative diagnoses were considered test negative. Equivocal cases on CT were also captured. Results of other additional tests performed, such as complete blood count (CBC) or urinalysis that were performed were captured, along with clinical findings that were used to calculate individual patient’s Alvarado scores.

Histological diagnoses for those who underwent appendectomy and a follow up of two weeks from the initial evaluation in outpatient clinic were considered the reference standard. Reference standard positive was defined histologically as the appendix with transmural presence of acute inflammatory cells. The reference standard negative was defined as specimens not fitting the histological definition for AA in those who underwent appendectomy or did not require an appendectomy after two weeks follow up.

Those with equivocal findings on the index test or those that did not undergo the reference standard were excluded in diagnostic performance analysis. Sensitivity, specificity, diagnostic accuracy, predictive values, and area under the receiver operator characteristics curve (AUROC) were determined for the index test of suspected AA. Analyses were conducted using the IBM SPSS version 25 Statistical package.

RESULTS

A total of 176 patient records were included in the study, of which 29.5 % (52/176) were females, and the mean age was 35 years (95% CI of 34-37). The vast majority (91.5%) had no pre-existing comorbidities. The mean duration from illness to emergency medicine department (EMD) presentation was 2 days (95% CI 1.8-2.3). At the EMD, the first assessor was a medical officer in 40.3%, surgical residents 36.4%, and a specialist in 23.3% of the cases.

All patients were assessed clinically and underwent a CBC. The mean computed AS was 4.64 (95% CI 4.32-4.97). Those with an AS of 1 to 4 were 50.6%, 5 to 8 were 43.2%, and greater than 8 were 6.3%. Urinalysis was done in 134 patients and was normal in 79.1% of these, 19.4% showing microscopic hematuria, and pyuria with leucocytes in 1.5%. The distribution of the demographic features is summarized in Table 1.

Table 1. Distribution of participants. demographic features (N=176)

	Frequency	Percentage (%)
	**Sex of Participants**	
**Males**	124	70.5
**Female**	52	29.5
	**Age Group in Years**	

**25 <**	31	17.6
**26-35**	59	33.5
**36-45**	56	31.8
**46 >**	30	17.0
	**Duration of Illness in Days**	
**1**	105	59.7
**2**	24	13.6
**3**	17	9.7
**4**	11	6.3
**>5**	19	10.8
	**Alvarado Score groups**	
**1 to 4**	89	50.6
**5 to 8**	76	43.2
**>8**	11	6.3
	**Comorbid**	
**Present**	15	8.5
**Absent**	161	91.5
	**Urinalysis**	
**Abnormal**	28	15.9
**Normal**	106	60.2
**Not done**	42	23.9
	**Designation of Assessor**	
**Registrar**	71	40.3
**Resident**	64	36.4
**Consultant**	41	23.3

All participants underwent CT investigation for suspected AA. Plain CTs were done in 87.5% of the patients, 10.2%, 1.7%, and 0.6% used intravenous, oral and rectal contrast, respectively. On CT scan AA was confirmed in 26.7% (47/176) of the cases, of which 17.0% (8/47) were complicated. Of those determined to have AA on CT and underwent appendectomy, 43 were confirmed histologically to have AA, while 4 were not.

The CT scan results were negative for AA in 70.4% (124/176). Of these, 77.4% (96/124) had alternative diagnoses. The commonest alternative diagnosis was urolithiasis in 70.8% (68/96). Of the remaining CT scan negative cases for AA without alternative diagnoses, 3 of 28 participants underwent appendectomy following the observation period and were histologically negative. The other 25 participants completed the 14-day outpatient follow-up and were declared not to have AA. The flow of participants is summarized in Figure 1 below.

To assess which features were associated with CT diagnosis of AA, binomial logistic regression analysis was done whereby calculated AS had a statistically significant association with CT diagnosis of AA. The mean AS in those without CT confirmed AA was 4 (95% CI 3.7-4.3) compared to mean AS of 6.6 (95% CI 6.0-7.2) among those with CT confirmed AA. In contrast, sex, age, urinalysis results or designation of the assessor did not have a statistically significant association.

Figure 1. Flow of participants

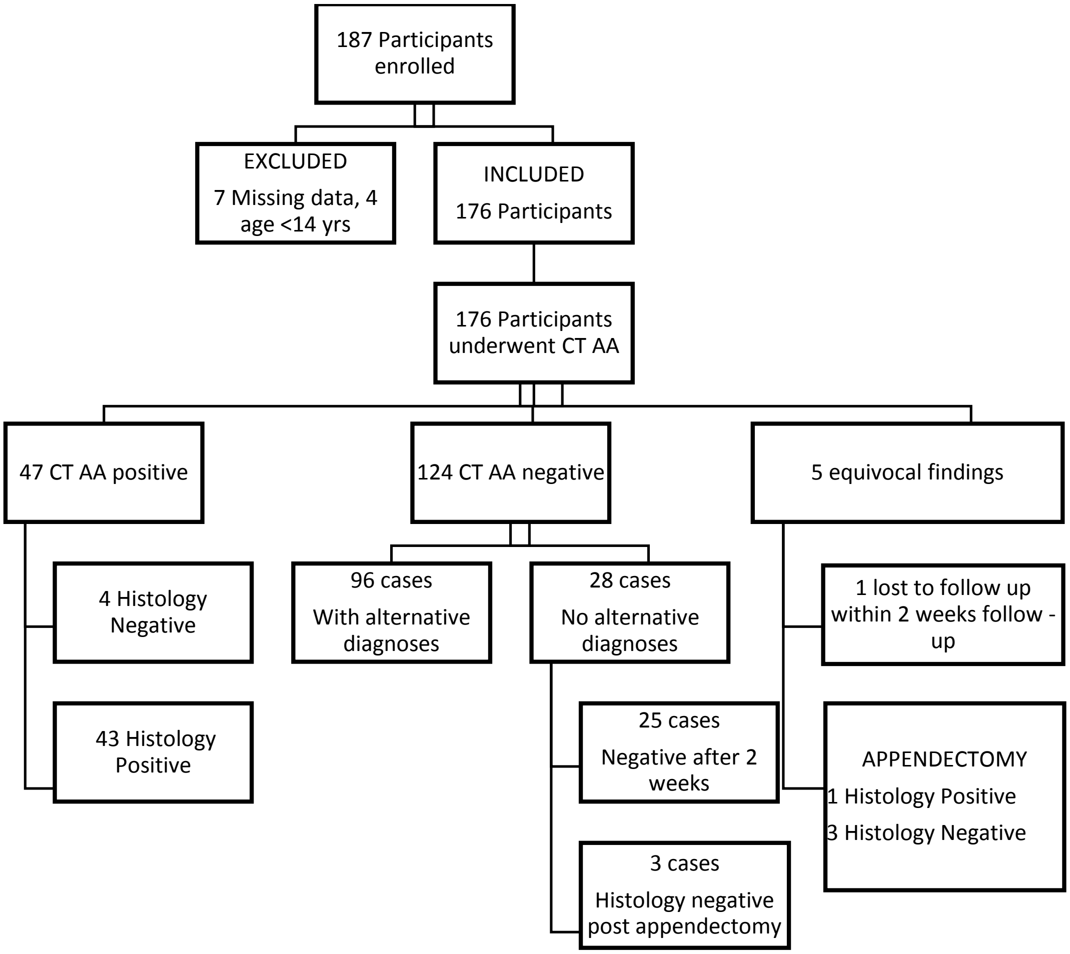

The prevalence of AA in this study was 25.1% (44/175). For diagnostic performance analysis, 171 participants were included, as 5 had equivocal findings. Cross-tabulation between CT and the reference standards is summarized in Table 2. The sensitivity, specificity, and diagnostic accuracy of CT for adults with suspected AA were 100% (95% CI 91.8-100), 96.9% (95% CI 92.2-99.1), and 96.9% (95% CI 93.1-98.3), respectively. Thus, at a prevalence of 25% in our study, the CT's positive and negative predictive values were 91.5% (95% CI 80.1 -96.6) and 100% (95% CI 97.0-100).

Table 2. Cross-tabulation between CT results and Reference standard

	REFERENCE STANDARD		
	**Negative**	**Positive**	
Index Test Negative	124	0	124
Index Test Positive	4	43	47
**Total**	128	43	171

ROC curve for the CT revealed an AUROC of 98.4%, and that of AS had an AUROC of 84.1%. A cut-off AS value of 4 had a sensitivity of 90.7% (95% CI 77-97), specificity of 65.6% (95% CI 56.7.1-73.7) and an NPV of 95.4% (95% CI 89-98), and was selected as a cut off to rule out AA. An AS of 8 had a specificity of 97% (95% CI 93-99) and a PPV of 84% (95% CI 61- 94).

The ROC curves are shown in figure 2.

Figure 2. Receiver Operator Characteristics Curve of CT and Alvarado Score

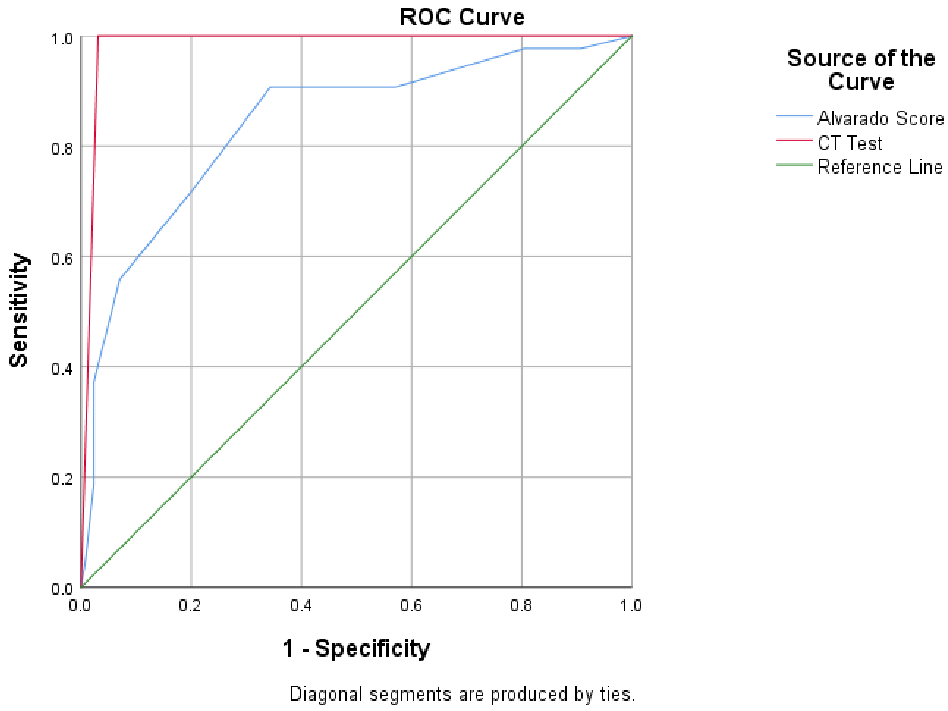

DISCUSSION

The contextual differences in SSA had led to the hypothesis that the diagnostic accuracy of CT for AA might be different. However, in this study, the diagnostic accuracy found is comparable to that demonstrated in metanalyses elsewhere (7-12). The excellent diagnostic performance of CT for suspected AA needs to be considered in the context of healthcare costs, length of stay at the EMD and adverse risk of radiation exposure (23, 24).

A large retrospective study evaluating the diagnostic accuracy of CT for suspected AA identified 106 patients who could have avoided CT by using AS for screening. The collective duration spent at the EMD from ordering to receiving the CT report for these patients was 10 239 hours (23). In another study over 5 years, the cumulative cost was 173,998 USDs for CT investigation that could have been avoided in males younger than 45 years presenting with classical features of AA (24). In the current study, 70% (124/176) of the CT investigations for suspected AA were negative,

raising the concern of whether this rate could have been lowered. An AS of 4 or less has sufficient sensitivity to rule out AA as concluded by international guidelines (25, 26). Using this AS cut-off as a threshold to conduct CT, 50% of patients could have avoided CT investigations and missed 4 cases of AA. There are no internationally agreed AS cut off to proceed to appendectomy, in this study using solely a cut off AS of 8 to conduct appendectomy without CT evaluation would have resulted in a negative appendectomy rate of 15% (3/19) that is considered unacceptable in the CT use era (21).

The AS was not documented in patients' charts in this study and was computed based on the collected data. Whether this resulted from attitudes and practices towards AS use for suspected AA could not be determined. However, it has been previously shown that AS is used in only 6% of suspected AA in this setting (27). Reasons for its uncommon use need to be determined and addressed to decrease CT overuse and negative appendectomy rates. To improve the cost-effective use of CT, we further emphasize the use of AS for screening all patients with suspected AA.

This study has some limitations; being a retrospective study, it relied on accurate documentation by the attending physicians and radiologists. Four independent radiologists interpreted the CT scans raising concern for inter-observer variability. The prevalence of AA in our study was 25%, instead of our sample size assumption of 43%. This lowered the precision of our results to 0.067, as opposed to our initial precision estimate of 0.05. At this precision of 0.067, the minimum sample size required is 163 participants.

CONCLUSION AND RECOMMENDATIONS

Overall, the diagnostic performance of CT demonstrated in this study is like that established outside of SSA, and the prevailing issue should be its selective use without adversely affecting AA

outcomes. The use of AS as a screening tool would increase the cost-effective use of CT. An AS of 4 should be used as a threshold to conduct CT. Implementation studies that would address the low use of AS should be conducted.

REFERENCES

1. Addiss DG, Shaffer N, Fowler BS, Tauxe RV. The epidemiology of appendicitis and appendectomy in the United States. Am J Epidemiol. 1990;132(5):910-25.
2. Ferris M, Quan S, Kaplan BS, Molodecky N, Ball CG, Chernoff GW, et al. The Global Incidence of Appendicitis: A Systematic Review of Population-based Studies. Ann Surg. 2017;266(2):237-41.
3. Murphy JB. " TWO THOUSAND OPERATIONS FOR APPENDICITIS," WITH DEDUCTIONS FROM HIS PERSONAL EXPERIENCE. The American Journal of the Medical Sciences (1827-1924). 1904;128(2):187.
4. Lameris W, van Randen A, Go PM, Bouma WH, Donkervoort SC, Bossuyt PM, et al. Single and combined diagnostic value of clinical features and laboratory tests in acute appendicitis. Acad Emerg Med. 2009;16(9):835-42.
5. Blomqvist PG, Andersson RE, Granath F, Lambe MP, Ekbom AR. Mortality after appendectomy in Sweden, 1987-1996. Ann Surg. 2001;233(4):455-60.
6. Jaunoo SS, Hale AL, Masters JP, Jaunoo SR. An international survey of opinion regarding investigation of possible appendicitis and laparoscopic management of a macroscopically normal appendix. Ann R Coll Surg Engl. 2012;94(7):476-80.
7. Yun SJ, Ryu CW, Choi NY, Kim HC, Oh JY, Yang DM. Comparison of Low- and Standard-Dose CT for the Diagnosis of Acute Appendicitis: A Meta-Analysis. AJR Am J Roentgenol. 2017;208(6):W198-W207.

8. Terasawa T, Blackmore CC, Bent S, Kohlwes RJ. Systematic review: computed tomography and ultrasonography to detect acute appendicitis in adults and adolescents. Ann Intern Med. 2004;141(7):537-46.
9. Anderson BA, Salem L, Flum DR. A systematic review of whether oral contrast is necessary for the computed tomography diagnosis of appendicitis in adults. Am J Surg. 2005;190(3):474-8.
10. Al-Khayal KA, Al-Omran MA. Computed tomography and ultrasonography in the diagnosis of equivocal acute appendicitis. A meta-analysis. Saudi Med J. 2007;28(2):173-80.
11. Hlibczuk V, Dattaro JA, Jin Z, Falzon L, Brown MD. Diagnostic accuracy of noncontrast computed tomography for appendicitis in adults: a systematic review. Ann Emerg Med. 2010;55(1):51-9 e1.
12. Rud B, Vejborg TS, Rappeport ED, Reitsma JB, Wille-Jorgensen P. Computed tomography for diagnosis of acute appendicitis in adults. Cochrane Database Syst Rev. 2019;2019(11).
13. Krajewski S, Brown J, Phang PT, Raval M, Brown CJ. Impact of computed tomography of the abdomen on clinical outcomes in patients with acute right lower quadrant pain: a meta-analysis. Can J Surg. 2011;54(1):43-53.
14. Aly OE, Black DH, Rehman H, Ahmed I. Single incision laparoscopic appendicectomy versus conventional three-port laparoscopic appendicectomy: A systematic review and meta-analysis. Int J Surg. 2016;35:120-8.
15. Dahabreh IJ, Adam GP, Halladay CW, Steele DW, Daiello LA, Wieland LS, et al. Diagnosis of right lower quadrant pain and suspected acute appendicitis. 2015.
16. Simba DO, Mbembati NA, Museru LM, Lema LE. Referral pattern of patients received at the national referral hospital: challenges in low income countries. East Afr J Public Health. 2008;5(1):6-9.
17. Mbembati NA, Mwangu M, Muhondwa EP, Leshabari MM. Performance indicators for quality in surgical and laboratory services at Muhimbili National Hospital (MNH) in Tanzania. East Afr J Public Health. 2008;5(1):13-6.
18. Hsia RY, Mbembati NA, Macfarlane S, Kruk ME. Access to emergency and surgical care in sub-Saharan Africa: the infrastructure gap. Health Policy Plan. 2012;27(3):234-44.

CC BY SA

19. Penoyar T, Cohen H, Kibatala P, Magoda A, Saguti G, Noel L, et al. Emergency and surgery services of primary hospitals in the United Republic of Tanzania. BMJ Open. 2012;2(1):e000369.

20. Yang E, Kahn D, Cook C. Acute appendicitis in South Africa: a systematic review. S Afr J Surg. 2015;53(3 and 4):31-8.

21. Tseng J, Cohen T, Melo N, Alban RF. Imaging utilization affects negative appendectomy rates in appendicitis: an ACS-NSQIP study. The American Journal of Surgery. 2019;217(6):1094-8.

22. Buderer NMF. Statistical methodology: I. Incorporating the prevalence of disease into the sample size calculation for sensitivity and specificity. Academic Emergency Medicine. 1996;3(9):895-900.

23. Coleman JJ, Carr BW, Rogers T, Field MS, Zarzaur BL, Savage SA, et al. The Alvarado score should be used to reduce emergency department length of stay and radiation exposure in select patients with abdominal pain. Journal of Trauma and Acute Care Surgery. 2018;84(6):946-50.

24. Westfall KM, Purcell LN, Charles AG. Computed Tomography for Acute Appendicitis Diagnosis and Confirmation in Men: Trends and Cost Implications. The American Surgeon. 2021;87(3):364-9.

25. Gorter RR, Eker HH, Gorter-Stam MA, Abis GS, Acharya A, Ankersmit M, et al. Diagnosis and management of acute appendicitis. EAES consensus development conference 2015. Surgical endoscopy. 2016;30(11):4668-90.

26. Di Saverio S, Birindelli A, Kelly MD, Catena F, Weber DG, Sartelli M, et al. WSES Jerusalem guidelines for diagnosis and treatment of acute appendicitis. World Journal of Emergency Surgery. 2016;11(1):1-25.

27. Nyamuryekung'E MK, Athar A, Patel MR, Njau A, Sherman O, JusabanI A, et al. Negative Appendectomy Rate in Urban Referral Hospitals in Tanzania: A Cross-sectional Analysis of Associated Factors. Annals of African Surgery. 2021;18(2):109-14.

STARD CHECKLIST

Section & Topic	No	Item	Reported on page #
**TITLE OR ABSTRACT**			
	**1**	Identification as a study of diagnostic accuracy using at least one measure of accuracy (such as sensitivity, specificity, predictive values, or AUC)	Page 1
**ABSTRACT**			
	**2**	Structured summary of study design, methods, results, and conclusions (for specific guidance, see STARD for Abstracts)	Page 2
**INTRODUCTION**			
	**3**	Scientific and clinical background, including the intended use and clinical role of the index test	Page 4
	**4**	Study objectives and hypotheses	Page 5
**METHODS**			
Study design	**5**	Whether data collection was planned before the index test and reference standard were performed (prospective study) or after (retrospective study)	Page 5
Participants	**6**	Eligibility criteria	Page 5
	**7**	On what basis potentially eligible participants were identified (such as symptoms, results from previous tests, inclusion in registry)	Page 5
	**8**	Where and when potentially eligible participants were identified (setting, location and dates)	Page 5
	**9**	Whether participants formed a consecutive, random or convenience series	Page 6
Test methods	**10a**	Index test, in sufficient detail to allow replication	Page 6
	**10b**	Reference standard, in sufficient detail to allow replication	Page 6
	**11**	Rationale for choosing the reference standard (if alternatives exist)	N/A
	**12a**	Definition of and rationale for test positivity cut-offs or result categories of the index test, distinguishing pre-specified from exploratory	Page 6
	**12b**	Definition of and rationale for test positivity cut-offs or result categories of the reference standard, distinguishing pre-specified from exploratory	Page 6
	**13a**	Whether clinical information and reference standard results were available to the performers/readers of the index test	Page 6
	**13b**	Whether clinical information and index test results were available to the assessors of the reference standard	Page 6
Analysis	**14**	Methods for estimating or comparing measures of diagnostic accuracy	Page 6
	**15**	How indeterminate index test or reference standard results were handled	Page 6
	**16**	How missing data on the index test and reference standard were handled	Page 6
	**17**	Any analyses of variability in diagnostic accuracy, distinguishing pre-specified from exploratory	N/A
	**18**	Intended sample size and how it was determined	Page 5
**RESULTS**			
Participants	**19**	Flow of participants, using a diagram	Page 10
	**20**	Baseline demographic and clinical characteristics of participants	Page 7
	**21a**	Distribution of severity of disease in those with the target condition	Page 8 & 9
	**21b**	Distribution of alternative diagnoses in those without the target condition	Page 8 & 9
	**22**	Time interval and any clinical interventions between index test and reference standard	Page 9
Test results	**23**	Cross tabulation of the index test results (or their distribution) by the results of the reference standard	Page 11
	**24**	Estimates of diagnostic accuracy and their precision (such as 95% confidence intervals)	Page 10
	**25**	Any adverse events from performing the index test or the reference standard	Not applicable
**DISCUSSION**			
	**26**	Study limitations, including sources of potential bias, statistical uncertainty, and generalisability	Page 13
	**27**	Implications for practice, including the intended use and clinical role of the index test	Page 12
**OTHER INFORMATION**			
	**28**	Registration number and name of registry	Page 7
	**29**	Where the full study protocol can be accessed	Plos data sharing policy
	**30**	Sources of funding and other support; role of funders	Author form

Diagnostic accuracy of computed tomography in adults with suspected acute appendicitis at the emergency department in a private tertiary hospital in Tanzania

Masawa K. Nyamuryekung'e[1*], Miten R. Patel[1¶], Ahmed Jusabani[1¶], Ali A. Zehri[1, #a, ¶], Athar Ali[1¶]

[1]Department of Surgery, The Aaga Khan Hospital, Dar es salaam, Tanzania

[#a]Current Address: Department of Surgery, Mukhtar A. Sheikh Hospital, Punjab, Pakistan

*Corresponding Author

Ee-mail: masawa4@hotmail.com (MKN)

[¶] These authors contributed equally to this work

Acknowledgments: We thank Kasusu Klint Nyamuryekung'e (DDS, MPhil Int. Health, PhD) for his assistance and inputs in the statistical analysis and preparation of the manuscript.

ABSTRACT

Introduction

The increasing incidence of acute appendicitis in sub-Saharan Africa emphasizes the need for accurate and reliable diagnostic tools. However, the ~~established wide ranges of~~variability in the diagnostic performance of computed tomography for suspected acute appendicitis, ~~in recent metanalyses, lack of local studies, and~~coupled with comparatively higher negative appendectomy rates in this setting highlight a possible concern ~~of accepting that~~ regarding the diagnostic accuracy. ~~of computed tomography for suspected acute appendicitis in this region is as high as that established in summary estimates.~~

This~~e current~~ study evaluated the diagnostic accuracy of a computed tomography scan for ~~acute appendicitis in patients with~~ suspected acute appendicitis at the emergency department in Tanzania.

Methods

A retrospective diagnostic accuracy study was conducted from~~between~~ July ~~2020~~ to~~and~~ October 2020. All ~~adult~~ patients above 14 years of age who presented at the emergency department with right iliac fossa abdominal pain of fewer than ten days and underwent computed tomography for suspected acute appendicitis were evaluated, and the Alvarado score was computed. Histological diagnosis and clinical follow-up of 14 days were considered the reference standard. Ethical clearance was sought from the Aga Khan University Ethical review committee.~~, AKU-ERC, EA, with a reference number of AKU/2020/0170/fb.~~

Results

176 patients were included in this study. The sensitivity, specificity, and diagnostic accuracy were 100% (95% CI 91.8-100), 96.9% (95% CI 92.2-99.1), and 96.9% (95% CI 93.1-98.3), respectively. Although the Alvarado score was not documented, collected data allowed its computation. The mean Alvarado score in those without acute appendicitis was 4 (95% C.I 3.7-4.3) compared to a mean score of 6.6 (95% CI 6.0-7.2), and this difference was statistically significant (P<0.05) amongst those with acute .appendicitis. The area under the receiver operator characteristics curve of computed tomography was 0.98.4%, and that of the Alvarado score was 0.84.1%. A cut-off Alvarado score of 3 was selected to rule out acute appendicitis.

Conclusions

Overall, theThe diagnostic performance of computed tomography demonstrated in this study is similar to similar to that established elsewhere. However, the Alvarado score is not routinely used for the initial screening of suspected acute appendicitis patients. that established outside of sub-Saharan Africa, and the prevailing issue should be selective use of this modality without adversely affecting outcomes. AThe use of AS threshold of Alvarado score of 4 as a guide to conduct computed tomography for suspected acute appendicitis as aw screening tool ould have decreased computed tomography use by 50%, and missed 4 cases.would increase the cost-effective use of computed tomography Implementation studies that address Alvarado score use should be conducted., and implementation studies that would address the use of the Alvarado score should be conducted.

Formatted: Highlight

List of Abbreviations

AA- Acute Appendicitis

AS- Alvarado Score

AUROC- Area Under the Receiver Operator Characteristics curve

CT- Computed Tomography

CI- Confidence interval

EMD- Emergency Medicine Department

LR+- Positive Likelihood ratio

LR- Negative Likelihood ratio

ROC- Receiver operator characteristics

SSA- Sub Saharan Africa

WSES- World society of emergency surgery

KEYWORDS: Acute appendicitis, CT abdomen, Sensitivity, Diagnostic accuracy, Africa

DIAGNOSTIC ACCURACY OF COMPUTED TOMOGRAPHY IN ADULTS~~ADULTS~~ WITH SUSPECTED ACUTE APPENDICITIS AT THE EMERGENCY DEPARTMENT IN PRIVATE TERTIARY HOSPITAL IN TANZANIA

INTRODUCTION

Acute appendicitis (AA) is the commonest cause of acute abdomen in adults that frequently requires emergency surgical intervention (1). AA has been viewed primarily as a western disease; however, current disease trends have suggested increasing incidence in sub-Saharan Africa (SSA), underscoring its growing~~increasing~~ significance (2).

~~The~~ An accurate diagnosis of ~~acute appendicitis~~ AA can be made clinically about 50% of the time.~~;~~ I~~i~~n the remaining instances,~~ making a clinical~~ diagnosis solely through clinical findings ~~alone~~ may

be challenging (3). In this scenario, diagnosis delays or misdiagnosisThere is an increased likelihood of diagnosis delays or misdiagnosis in this scenario. This may result in higher will increase the appendicular perforation rate and negative appendectomy rates , which are comparatively higher in Africa (4, 5). To improve the diagnostic accuracy of AA, medical imaging tools are frequently utilized inis additionally to clinical findings. utilized. The Aabdominal computed tomography (CT) is the commonest preferred medical imaging used for this purpose in nonpregnant adults in the west (6). It

This modality allows for visualization visualization of the appendix, and evaluation of features known to predict AA . Other benefits include and determining alternative pathologies (7, 8).

Multiple systematic reviews and meta-analyses have established that CT's diagnostic accuracy for suspected AAacute appendicitis in nonpregnant adult patients is over 92% (7-12). However, in the pooled meta-analysis studies, the range of sensitivity and specificity of CT's diagnosis for suspected AA was 72 to 100% and 50 to 100%, respectively (12). There is further vVariability of diagnostic accuracy may be due to radiologists' experience, contrast use, differing CT generations, imaging protocols, and acute appendicitis prevalence in the study settings (7, 9, 11, 12).

In a systematic review and meta-analysis conducted in 2019 evaluating the diagnostic performance of CT in adults with suspected AA, the summary sensitivity was 95% (95% Confidence Interval 0.93 to 0.96). The summary specificity was 94% (95% Confidence Interval 0.92 to 0.95). However, the sensitivity range was from 0.72 to 1.00, and the specificity ranged from 0.5 to 1.00 .

There have been over ten meta-analyses and systematic reviews conducted to date assessing the diagnostic performance of CT scans for suspected acute appendicitisAA in adults, and none

include findings have studies from AfricaSSA, possibly due to the lack of eligible studies from this area (7-15). This partly reflects the limited resources of health facilities in terms of equipment available, expertise, experience, and possibly the preference of the imaging modality for suspected of AAacute appendicitis (16-19). Assessing tThe outcomes of performed appendectomies in SSAacute appendicitis reveal a negative appendectomy rate, the negative appendectomy rate should be lower with improved diagnostic capacity; in Africa, this is about of about 17%-, compared to 1- 5% in other areas, raising concern for diagnostic accuracy of suspected AA in this region (20, 21).

Due to the factors mentioned above, namely, the increasing incidence of AA in SSA, the established wide ranges of diagnostic performance of CT for suspected AA in recent metanalyses, lack of local studies, and higher negative appendectomy rates in SSA highlight a possible concern of accepting that CT's diagnostic accuracy for suspected AA in this region is as high as that established in summary estimates, and needs to be validated in this context.

The current study's mainmain objectives wereare to determine the sensitivity, specificity, diagnostic accuracy, and predictive values of CT for patients with suspected acute appendicitis AA presenting to the emergency medicine departmentdepartment (EMD) of Aga Khan Hospital, Tanzania.

METHODSOLOGY

This Ethical clearance for the study protocol was sought from the Aga Khan University Ethical review committee, AKU-ERC, EA, with a reference number of AKU/2020/0170/fb. Consent was waived as this was a retrospective chart review and the data collected was de identified. The study was conducted from July to October 2020 at a private tertiary teaching hospital in Dar es Salaam,

Tanzaniaretrospective diagnostic accuracy study was conducted from July 2020 to October 2020 at a private tertiary teaching hospital. Records of offering emergency medicine and surgical services with fully-fledged radiology and pathology department equipped with advanced medical imaging, including CT scans. pAdult patients agedfrom 14 years and above of age were included in the study, who presented at the emergency departmentEMD with right iliac fossa colicky abdominal pain for lessof fewer than 10 days and underwent CT abdomen for suspected AA were included in this study. Pregnant women and those who underwent abdominal-pelvic ultrasonography prior to the abdominalbefore CT scan were excluded. Sample size estimation based on the Buderer formula of diagnostic accuracy for unknown disease prevalence was employed (22). The assumptions applied were an acceptable marginal error of 0.05, hypothesized sensitivity of 0.95, specificity of 0.94 and prevalence of 0.43, based on Bo Rud, 2019 et al. (12). The required sample size for sensitivity and specificity were 170 and 153 participants, respectively. Consecutive sampling was utilized to include patient records into the study until the sample size was reached.patients meeting the inclusion and exclusion criteria were included until achieving sample size.

The index test for suspected AA was a helical CT scan, manufactured by Philips, Ingenuity model, 128 slices with 64 detector rows. One of four consultant medical radiologists interpreted the images individually during their on-call schedule. All radiologists had at least trained as Master of Medicine in medical radiology. The target condition was AA, encompassing both simple and complicated presentations. All cases diagnosed by CT to have simple or complicated AA were considered testTest positive. were all cases diagnosed by CT to have simple or complicated AA Alternately, all cases with a normal appendix on CT evaluation or alternative diagnoses were considered test negative. Test negative were all cases with a normal appendix on CT evaluation

~~and those with alternative diagnoses.~~ Equivocal cases on CT were also captured. Results of other additional tests performed, such as complete blood count (CBC) or urinalysis that were performed were captured, along with clinical findings that were used to calculate individual patient's Alvarado scores.~~.~~

Histological diagnoses for those who underwent appendectomy and a follow up of two weeks from the initial evaluation in outpatient clinic were considered ~~a~~ the reference standard. Reference standard positive~~AA~~ was defined histologically as the appendix with transmural presence~~attendance~~ of acute inflammatory cells~~. For patients who did not undergo appendectomy, subsequent 14 days after CT evaluation in the outpatient follow up was used as a reference standard~~. The reference standard negative was defined as~~all~~ ~~histological appendix~~ specimens not fitting the histological definition for AA in those who underwent ~~appendectom~~appendectomy~~y~~ or did not require an appendectomy after two weeks follow up.~~.~~

~~Sample size estimation based on Buderer formula of diagnostic accuracy for unknown disease prevalence , with the following assumptions, was used; an acceptable marginal error of 0.05, hypothesized sensitivity of 0.95, specificity of 0.94 and prevalence of 0.43, based on findings from Bo Rud, 2019 et al. . The required sample size for sensitivity is 170 participants and, for specificity, 153 participants. Sensitivity, specificity, predictive values, AUROC, and diagnostic accuracy were determined. Analyses were conducted using the IBM SPSS version 25 Statistical package.~~

Formatted: Highlight

~~The study was a chart review of retrospectively collected data with no direct patient contact. The use of codes for identification ensured confidentiality and anonymity.~~Those with equivocal findings on the index test or those that did not undergo the reference standard were excluded in

diagnostic performance analysis. Sensitivity, specificity, diagnostic accuracy, predictive values, and area under the receiver operator characteristics curve (AUROC) were determined for the index test of suspected AA. Analyses were conducted using the IBM SPSS version 25 Statistical package. ~~Ethical clearance for the study protocol was sought from the Aga Khan University Ethical review committee, AKU-ERC, EA, with a reference number of AKU/2020/0170/fb.~~

RESULTS

A total of 176 ~~Participants~~ patient records were included in the study, of which ~~comprised~~ 29.5 % (52/176) were females, ~~with~~ and the mean ~~participants'~~ age was 35 years (95% CI of 34-37). Th~~os~~e ~~without comorbidities constituted~~vast majority (-91.5%) had no pre-existing comorbidities. ~~of the participants, 5.7% had hypertension, 1.1% diabetes, and 1.7% had other comorbidities.~~ The mean duration from~~of~~ illness to ~~presentation to the~~ emergency medicine department (EMD) presentation was 2 days (95% CI 1.8-2.3)~~), 59.7 % of the participants presented to the hospital accidents and emergency department on the first day of illness; by day second, third and fourth days, the cumulative percentages were 73.3%, 83.0% and 89.2%, respectively~~. At the EMD, the first assessor was a medical officer in 40.3%, surgical residents 36.4%, and a specialist in 23.3% of the cases.

All patients were assessed clinically and underwent a CBC.~~,~~ ~~The Alvarado score (AS) was not documented. However, based on symptoms assessed and the results of the CBC, the AS was computed for each patient.~~ The mean computed AS was 4.64 (95% CI 4.32-4.97).~~,~~ T~~t~~hose with an AS of 1 to 4 were 50.6%, 5 to 8 were 43.2%, and greater than 8 were 6.3%.

Formatted: Highlight

~~In addition to symptoms captured in the AS, 5.1% had urinary frequency, 1.1% nocturia and 7.4% dysuria.~~ Urinalysis was done in 134 patients and was normal in 79.1~~60.2~~% of these, 19.4%

showing microscopic hematuria, ~~in 14. 8%, pyuria~~ and pyuria with leucocytes in 1.5~~1~~%. The distribution of the demographic features is ~~summarized~~ summarized in Table 1.

Table 1. Distribution of participants. demographic features (N=176)

	Frequency	Percentage (%)
**Sex of Participants**		
**Males**	124	70.5
**Female**	52	29.5
~~**Total**~~	~~**176**~~	~~**100.0**~~
**Age Group in Years**		
**25 <**	31	17.6
**26-35**	59	33.5
**36-45**	56	31.8
**46 >**	30	17.0
~~**Total**~~	~~**176**~~	~~**100.0**~~
**Duration ~~if~~ of Illness in Days**		
**1**	105	59.7
**2**	24	13.6
**3**	17	9.7
**4**	11	6.3
**>5**	19	10.8
~~**Total**~~	~~**176**~~	~~**100.0**~~
**Alvarado Score groups**		
**1 to 4**	89	50.6
**5 to 8**	76	43.2
**>8**	11	6.3
~~**Total**~~	~~**176**~~	~~**100.0**~~
**~~Comorbids~~Comorbid**		
**Present**	15	8.5
**Absent**	161	91.5
~~**Total**~~	~~**176**~~	~~**100.0**~~
**Urinalysis**		
**Abnormal**	28	15.9

Formatted Table

Formatted Table

Formatted Table

Formatted Table

Formatted: Font: Bold

Formatted Table

**Normal**	106	60.2
**Not done**	42	2~~3~~3.9
~~**Total**~~	~~**176**~~	~~**100.0**~~
**Designation of Assessor**		
**Registrar**	71	40.3
**Resident**	64	36.4
**Consultant**	41	23.3
~~**Total**~~	~~**176**~~	~~**100.0**~~

Formatted Table

All participants underwent CT investigation for suspected AA.~~;~~ P~~p~~lain CTs were done in 87.5% of the patients, 10.2%, ~~% using intravenous,~~ 1.7%~~,~~ ,~~oral,~~ and 0.6% used intravenous, oral and rectal contrast, respectively. On CT scan AA was ~~found~~confirmed in 26.7% (47/176)~~,~~ of the cases, of which 17.0% (8/47) were complicated~~, and 83.0% (39/47) were non-complicated.~~. Of those determined to have AA on CT and underwent appendectomy, 43 were confirmed histologically to have AA, while 4 were not.

The CT scan results were ~~equivocal in 2.8% (5/176) and were~~ negative for AA in 70.4% (124/176). Of these, ~~Among negative CT for AA,~~ 77.4% (96/124) had alternative diagnoses~~,~~. ~~and 22.6% (28/124) had no alternative diagnoses.~~ The commonest alternative diagnosis was urolithiasis in 70.8% (68/96). Of the remaining CT scan negative cases for AA without alternative diagnoses, 3 of 28 participants underwent appendectomy following the observation period and were histologically negative. The other 25 participants completed the 14-day outpatient follow-up and were declared not to have AA. The flow of participants is summarized in Figure 1 below.

To assess which features were associated with CT diagnosis of AA, binomial logistic regression analysis was done whereby calculated AS had a statistically significant association with CT diagnosis of AA~~ (P< 0.05)~~. The mean AS in those without CT confirmed AA was 4 (95% C~~.~~I 3.7-4.3) compared to mean AS of 6.6 (95% CI 6.0-7.2) among those with CT confirmed AA~~, and this difference was statistically significant in independent samples t-test (P<0.05)~~. In contrast, sex, age, urinalysis results or designation of the assessor did not have a statistically significant ~~n~~ association ~~(P>0.05)~~.

~~All 28 participants with normal appendix and no alternative diagnoses and 5 with equivocal features on CT were admitted for a 24-hour serial clinical assessment. 4 participants with equivocal findings underwent appendectomy, whereby 1 had histologically confirmed AA and 3 did not. The fifth participant with equivocal findings on CT was discharged after the observation period and was lost to follow-up. 3 of the 28 participants without AA on CT underwent appendectomy following the observation period and were histologically negative. The other 25 participants completed the 14- day outpatient follow-up and were declared not to have AA. Of those determined to have AA on CT and underwent appendectomy, 43 were confirmed histologically to have AA, while 4 did not.~~

~~The reference standard was applied to 175 participants, as 1 patient with equivocal findings on CT was lost to follow up. To determine the diagnostic performance of CT, the 5 patients with equivocal CT diagnoses were excluded from the analysis. The flow of participants is summarized in Figure 1 below.~~

Figure 1. Flow of participants

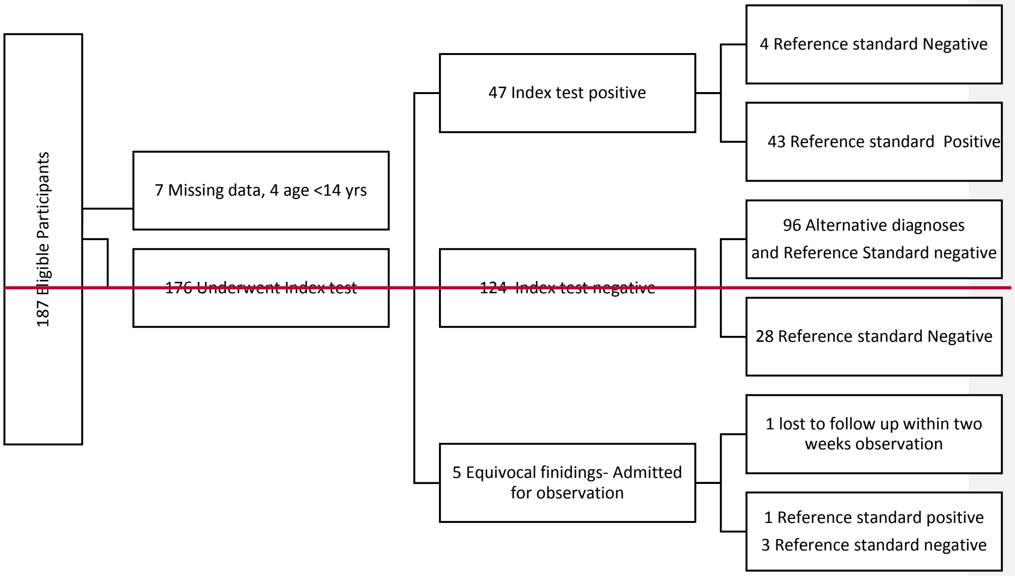
187 Eligible Participants
7 Missing data, 4 age <14 yrs
176 Underwent Index test
47 Index test positive
124 Index test negative
5 Equivocal finidings- Admitted for observation
4 Reference standard Negative
43 Reference standard Positive
96 Alternative diagnoses and Reference Standard negative
28 Reference standard Negative
1 lost to follow up within two weeks observation
1 Reference standard positive
3 Reference standard negative

162

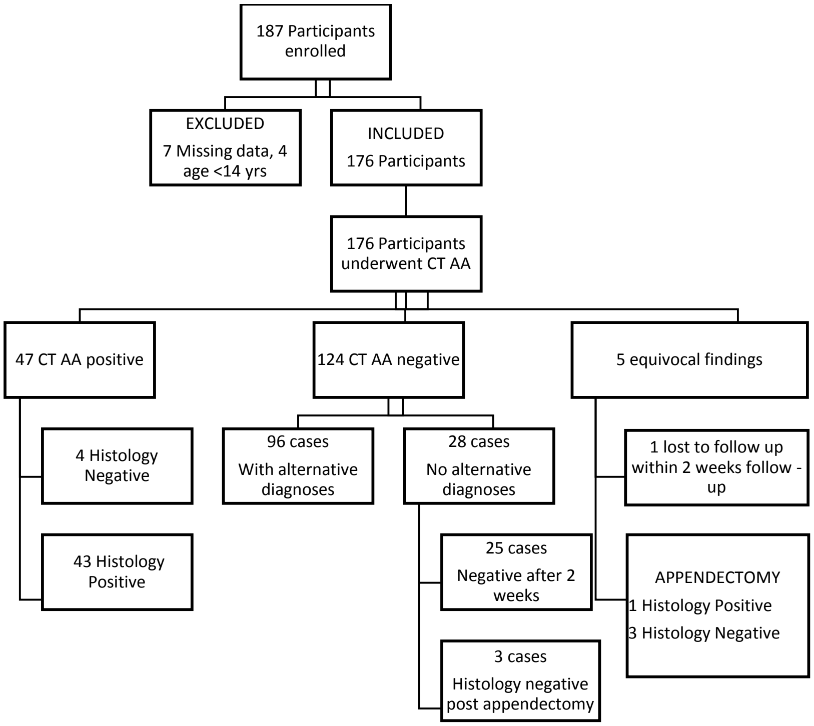

The prevalence of AA in this study~~histologically~~ was 25.1% (43~~43~~/175~~171~~). For diagnostic performance analysis, 171 participants were included, as 5 had equivocal findings. Cross-tabulation between CT and the reference standards is ~~summarized~~ summarized in Table 2. The sensitivity, specificity, and diagnostic accuracy of CT for adults with suspected AA were 100% (95% CI 91.8-100), 96.9% (95% CI 92.2-99.1), and 96.9% (95% CI 93.1-98.3), respectively. Thus, at a prevalence of 25% in our study, the CT's positive and negative predictive values were 91.5% (95% CI 80.1 -96.6) and 100% (95% CI 97.0-100).

Table 2. Cross-tabulation between CT results and Reference standard

	REFERENCE STANDARD		
	**Negative**	**Positive**	
IndexCT Test Negative	124	0	124
Index TestCT Positive	4	43	47
**Total**	128	43	171

ROC curve for the CT revealed an AUROC of 0.98.4%., and that of AS had an AUROC of 0.84.1%. A cut-off AS value of 43 had a sensitivity of 90.7% (95% C.I 776-976), specificity of 6525.61% (95% C.I 56,725.1-73,327.4) and an NPV of 953.42% (95% C.I 893-988), and was selected as a cut off to rule out AA. An AS of 8 had a specificity of 97% (95% CI 93-99) and a PPV of 84% (95% CI 61- 94).

The ROC curves are shown in figure 2.

Figure 2. Receiver Operator Characteristics Curve of CT and Alvarado Score

Formatted Table

Formatted: Indent: Left: -0.09"

Formatted: Not Highlight

Formatted: Not Highlight

Formatted: Not Highlight

Formatted: Not Highlight

Formatted: Not Highlight

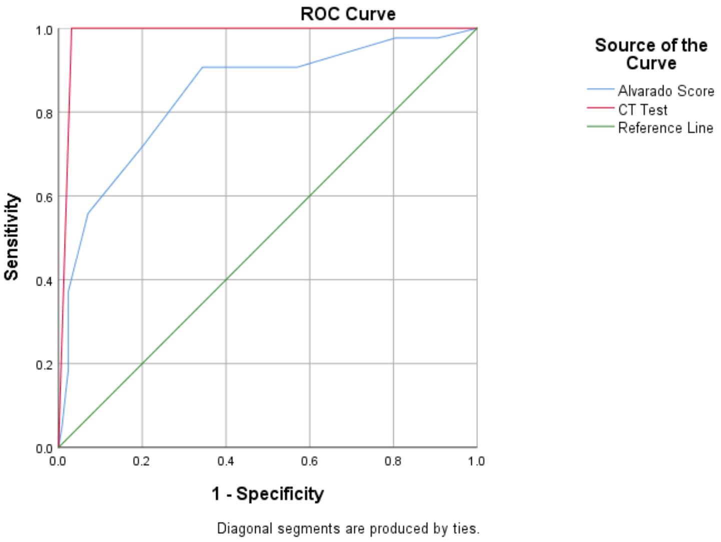

DISCUSSION

The contextual differences in SSA had led to the hypothesis that the diagnostic accuracy of CT for AA might be different. However, ~~The diagnostic accuracy of CT for suspected AA~~ in this study, the diagnostic accuracy found is ~~is~~ comparable to that demonstrated in metanalyses elsewhere~~outside of SSA~~ (7-12). The excellent diagnostic performance of CT for suspected AA needs to be considered in the context of healthcare costs, length of stay at the EMD and adverse risk of radiation exposure (23, 24).

Formatted: Not Highlight

A large retrospective study evaluating the diagnostic accuracy of CT for suspected AA identified 106 patients who could have avoided CT by using AS for screening. The collective duration spent at the EMD from ordering to receiving the CT report for these patients was 10 239 hours (23). In another study over 5 years, the cumulative cost was 173,998 USDs for CT investigation that could have been avoided in males younger than 45 years presenting with classical features of AA (24).

The contextual differences in SSA had led to the hypothesis that the diagnostic accuracy might be different; The demonstrated results do not show this difference, further emphasizing that the CT results are objective . The predictive values were similar to those described in other settings .

Formatted: Highlight

Despite the excellent diagnostic accuracy of CT for AA, thereIn the current study, is a significant concern about its overuse. In this study, 70% (124/176) of the CT investigations for suspected AA were negative, raising the concern of whether this rate could have been lowered. A similar negative CT result of 70% in the evaluation of adults with suspected AA was found in a large retrospective study, and concerns for cost implications, length of stay at the EMD, and the adverse risk of unnecessary radiation exposure were discussed, and use of AS was proposed to address the concerns .

An AS of 4 or less The AShas sufficient sensitivity to rule out AA as concluded by international guidelines has been shown to have utility in determining those who do not have AA. In the WSES guidelines, a score of less than 4 was deemed sufficiently sensitive to rule out AA, and no AS cut-off was sufficiently specific to rule in AA (25, 26). Using this AS In this study, we proposed a cut-off AS of as a3 to rule out AA. Using this threshold to notto conduct CT for suspected AA, CT use would have decreased50% of patients could have avoided CT investigations from 176 to 117 and missed 4 cases of AA. There are no internationally agreed AS cut off to proceed to appendectomy, in this study using solely a cut off AS of 8 to conduct appendectomy without CT evaluation would have resulted in a negative appendectomy rate of 15% (3/19) that is considered unacceptable in the CT use era (21).

The AS was not documented in patients' charts in this study and was computed based on the collected data. Whether this resulted from attitudes and practices towards AS use for suspected

AA could not be determined. However, ~~Surprisingly, i~~it has been previously shown that AS is used in only 6% of suspected AA in this~~our~~ setting (27). Reasons for its uncommon use need to be determined and addressed to decrease CT overuse~~ and negative appendectomy rate~~ and negative appendectomy rates.~~.~~

~~Correct elicitation of the AS is critical to realize its diagnostic performance fully. In this study, urolithiasis led to 37% of CT tests for suspected AA. This rate could have been lowered by the accurate elicitation of clinical features of AS. The presence of lower quadrant abdominal pain, rigidity and migratory abdominal pain have been shown to have LR+ 8.0, 4.0 and 3.1, respectively. The absence of right lower quadrant pain and classical migratory abdominal pain have been shown to have LR- of 0.2 and 0.5, respectively .~~

Formatted: Highlight

To improve the cost-effective use of CT, we further emphasize~~recommend~~ ~~regular and accurate~~ the use of~~elicitation of~~ AS for screening all patients with suspected AA.~~. Those with a score of less than 3 are unlikely to have AA, and alternative diagnoses should be considered. For a AS higher than 3, admission and serial clinical evaluation or CT imaging should be considered.~~

Formatted: Highlight

This study has some limitations; being a~~'s~~ retrospective study, it relied on accurate documentation by the attending physicians and radiologists. Four independent radiologists interpreted the CT scans raising concern for inter-observer variability.~~nature might have been a source of confirmation bias and is a possible limitation.~~ T~~In addition, t~~he prevalence of AA in our study was 25%, ~~as opposed to~~instead of our sample size assumption of 43%. This lowered the precision of our results to 0.067, as opposed to our initial precision estimate of 0.05. At this precision of 0.067, the minimum sample size required is 163 participants.

CONCLUSION AND RECOMMENDATIONS

Overall, the diagnostic performance of CT demonstrated in this study is ~~similar to~~like that established outside of SSA, and the prevailing issue should be its selective use ~~of this modality~~ without adversely affecting AA outcomes. The use of AS as a screening tool would increase the cost-effective use of CT. An AS of 4 should be used as a threshold to conduct CT.~~, and i~~Implementation studies that would address the low use of AS should be conducted.

~~In conclusion, during the initial evaluation of adults with suspected AA, AS should be done as a screening tool, and in select cases with AS >3, the use of CT is beneficial.~~

REFERENCES

1. Addiss DG, Shaffer N, Fowler BS, Tauxe RV. The epidemiology of appendicitis and appendectomy in the United States. Am J Epidemiol. 1990;132(5):910-25.
2. Ferris M, Quan S, Kaplan BS, Molodecky N, Ball CG, Chernoff GW, et al. The Global Incidence of Appendicitis: A Systematic Review of Population-based Studies. Ann Surg. 2017;266(2):237-41.
3. Murphy JB. " TWO THOUSAND OPERATIONS FOR APPENDICITIS," WITH DEDUCTIONS FROM HIS PERSONAL EXPERIENCE. The American Journal of the Medical Sciences (1827-1924). 1904;128(2):187.
4. Lameris W, van Randen A, Go PM, Bouma WH, Donkervoort SC, Bossuyt PM, et al. Single and combined diagnostic value of clinical features and laboratory tests in acute appendicitis. Acad Emerg Med. 2009;16(9):835-42.
5. Blomqvist PG, Andersson RE, Granath F, Lambe MP, Ekbom AR. Mortality after appendectomy in Sweden, 1987-1996. Ann Surg. 2001;233(4):455-60.

Formatted: Justified, Indent: Hanging: 0.5", Line spacing: Double

Formatted: Font: (Default) Times New Roman, 12 pt

6. Jaunoo SS, Hale AL, Masters JP, Jaunoo SR. An international survey of opinion regarding investigation of possible appendicitis and laparoscopic management of a macroscopically normal appendix. Ann R Coll Surg Engl. 2012;94(7):476-80.
7. Yun SJ, Ryu CW, Choi NY, Kim HC, Oh JY, Yang DM. Comparison of Low- and Standard-Dose CT for the Diagnosis of Acute Appendicitis: A Meta-Analysis. AJR Am J Roentgenol. 2017;208(6):W198-W207.
8. Terasawa T, Blackmore CC, Bent S, Kohlwes RJ. Systematic review: computed tomography and ultrasonography to detect acute appendicitis in adults and adolescents. Ann Intern Med. 2004;141(7):537-46.
9. Anderson BA, Salem L, Flum DR. A systematic review of whether oral contrast is necessary for the computed tomography diagnosis of appendicitis in adults. Am J Surg. 2005;190(3):474-8.
10. Al-Khayal KA, Al-Omran MA. Computed tomography and ultrasonography in the diagnosis of equivocal acute appendicitis. A meta-analysis. Saudi Med J. 2007;28(2):173-80.
11. Hlibczuk V, Dattaro JA, Jin Z, Falzon L, Brown MD. Diagnostic accuracy of noncontrast computed tomography for appendicitis in adults: a systematic review. Ann Emerg Med. 2010;55(1):51-9 e1.
12. Rud B, Vejborg TS, Rappeport ED, Reitsma JB, Wille-Jorgensen P. Computed tomography for diagnosis of acute appendicitis in adults. Cochrane Database Syst Rev. 2019;2019(11).
13. Krajewski S, Brown J, Phang PT, Raval M, Brown CJ. Impact of computed tomography of the abdomen on clinical outcomes in patients with acute right lower quadrant pain: a meta-analysis. Can J Surg. 2011;54(1):43-53.
14. Aly OE, Black DH, Rehman H, Ahmed I. Single incision laparoscopic appendicectomy versus conventional three-port laparoscopic appendicectomy: A systematic review and meta-analysis. Int J Surg. 2016;35:120-8.
15. Dahabreh IJ, Adam GP, Halladay CW, Steele DW, Daiello LA, Wieland LS, et al. Diagnosis of right lower quadrant pain and suspected acute appendicitis. 2015.
16. Simba DO, Mbembati NA, Museru LM, Lema LE. Referral pattern of patients received at the national referral hospital: challenges in low income countries. East Afr J Public Health. 2008;5(1):6-9.

17. Mbembati NA, Mwangu M, Muhondwa EP, Leshabari MM. Performance indicators for quality in surgical and laboratory services at Muhimbili National Hospital (MNH) in Tanzania. East Afr J Public Health. 2008;5(1):13-6.
18. Hsia RY, Mbembati NA, Macfarlane S, Kruk ME. Access to emergency and surgical care in sub-Saharan Africa: the infrastructure gap. Health Policy Plan. 2012;27(3):234-44.
19. Penoyar T, Cohen H, Kibatala P, Magoda A, Saguti G, Noel L, et al. Emergency and surgery services of primary hospitals in the United Republic of Tanzania. BMJ Open. 2012;2(1):e000369.
20. Yang E, Kahn D, Cook C. Acute appendicitis in South Africa: a systematic review. S Afr J Surg. 2015;53(3 and 4):31-8.
21. Tseng J, Cohen T, Melo N, Alban RF. Imaging utilization affects negative appendectomy rates in appendicitis: an ACS-NSQIP study. The American Journal of Surgery. 2019;217(6):1094-8.
22. Buderer NMF. Statistical methodology: I. Incorporating the prevalence of disease into the sample size calculation for sensitivity and specificity. Academic Emergency Medicine. 1996;3(9):895-900.
23. Coleman JJ, Carr BW, Rogers T, Field MS, Zarzaur BL, Savage SA, et al. The Alvarado score should be used to reduce emergency department length of stay and radiation exposure in select patients with abdominal pain. Journal of Trauma and Acute Care Surgery. 2018;84(6):946-50.
24. Westfall KM, Purcell LN, Charles AG. Computed Tomography for Acute Appendicitis Diagnosis and Confirmation in Men: Trends and Cost Implications. The American Surgeon. 2021;87(3):364-9.
25. Gorter RR, Eker HH, Gorter-Stam MA, Abis GS, Acharya A, Ankersmit M, et al. Diagnosis and management of acute appendicitis. EAES consensus development conference 2015. Surgical endoscopy. 2016;30(11):4668-90.
26. Di Saverio S, Birindelli A, Kelly MD, Catena F, Weber DG, Sartelli M, et al. WSES Jerusalem guidelines for diagnosis and treatment of acute appendicitis. World Journal of Emergency Surgery. 2016;11(1):1-25.
27. Nyamuryekung'E MK, Athar A, Patel MR, Njau A, Sherman O, JusabanI A, et al. Negative Appendectomy Rate in Urban Referral Hospitals in Tanzania: A Cross-sectional Analysis of Associated Factors. Annals of African Surgery. 2021;18(2):109-14.

Formatted: Indent: First line: 0"

STARD CHECKLIST

Section & Topic	No	Item	Reported on page #
TITLE OR ABSTRACT			
	1	Identification as a study of diagnostic accuracy using at least one measure of accuracy (such as sensitivity, specificity, predictive values, or AUC)	Page 1
ABSTRACT			
	2	Structured summary of study design, methods, results, and conclusions (for specific guidance, see STARD for Abstracts)	Page 2
INTRODUCTION			
	3	Scientific and clinical background, including the intended use and clinical role of the index test	Page 4
	4	Study objectives and hypotheses	Page 5
METHODS			
Study design	5	Whether data collection was planned before the index test and reference standard were performed (prospective study) or after (retrospective study)	Page 5
Participants	6	Eligibility criteria	Page 5
	7	On what basis potentially eligible participants were identified (such as symptoms, results from previous tests, inclusion in registry)	Page 5
	8	Where and when potentially eligible participants were identified (setting, location and dates)	Page 5
	9	Whether participants formed a consecutive, random or convenience series	Page 6
Test methods	10a	Index test, in sufficient detail to allow replication	Page 6
	10b	Reference standard, in sufficient detail to allow replication	Page 6
	11	Rationale for choosing the reference standard (if alternatives exist)	N/A
	12a	Definition of and rationale for test positivity cut-offs or result categories of the index test, distinguishing pre-specified from exploratory	Page 6
	12b	Definition of and rationale for test positivity cut-offs or result categories of the reference standard, distinguishing pre-specified from exploratory	Page 6
	13a	Whether clinical information and reference standard results were available to the performers/readers of the index test	Page 6
	13b	Whether clinical information and index test results were available to the assessors of the reference standard	Page 6
Analysis	14	Methods for estimating or comparing measures of diagnostic accuracy	Page 6
	15	How indeterminate index test or reference standard results were handled	Page 6
	16	How missing data on the index test and reference standard were handled	Page 6
	17	Any analyses of variability in diagnostic accuracy, distinguishing pre-specified from exploratory	N/A
	18	Intended sample size and how it was determined	Page 5
RESULTS			
Participants	19	Flow of participants, using a diagram	Page 10
	20	Baseline demographic and clinical characteristics of participants	Page 7
	21a	Distribution of severity of disease in those with the target condition	Page 8 & 9
	21b	Distribution of alternative diagnoses in those without the target condition	Page 8 & 9
	22	Time interval and any clinical interventions between index test and reference standard	Page 9
Test results	23	Cross tabulation of the index test results (or their distribution) by the results of the reference standard	Page 11
	24	Estimates of diagnostic accuracy and their precision (such as 95% confidence intervals)	Page 10
	25	Any adverse events from performing the index test or the reference standard	Not applicable

cc BY SA

**DISCUSSION**			
	**26**	Study limitations, including sources of potential bias, statistical uncertainty, and generalisability	Page 13
	**27**	Implications for practice, including the intended use and clinical role of the index test	Page 12
**OTHER INFORMATION**			
	**28**	Registration number and name of registry	Page 7
	**29**	Where the full study protocol can be accessed	Plos data sharing policy
	**30**	Sources of funding and other support; role of funders	Author form

Section & Topic	No	Item	Reported on page #
**TITLE OR ABSTRACT**			
	**1**	Identification as a study of diagnostic accuracy using at least one measure of accuracy<br>(such as sensitivity, specificity, predictive values, or AUC)	Page 1
**ABSTRACT**			
	**2**	Structured summary of study design, methods, results, and conclusions<br>(for specific guidance, see STARD for Abstracts)	Page 2
**INTRODUCTION**			
	**3**	Scientific and clinical background, including the intended use and clinical role of the index test	Page 4
	**4**	Study objectives and hypotheses	Page 5
**METHODS**			
Study design	**5**	Whether data collection was planned before the index test and reference standard were performed (prospective study) or after (retrospective study)	Page 6
Participants	**6**	Eligibility criteria	Page 6
	**7**	On what basis potentially eligible participants were identified<br>(such as symptoms, results from previous tests, inclusion in registry)	Page 6
	**8**	Where and when potentially eligible participants were identified (setting, location and dates)	Page 6
	**9**	Whether participants formed a consecutive, random or convenience series	Page 6
Test methods	**10a**	Index test, in sufficient detail to allow replication	Page 6
	**10b**	Reference standard, in sufficient detail to allow replication	Page 6 & 7
	**11**	Rationale for choosing the reference standard (if alternatives exist)	N/A
	**12a**	Definition of and rationale for test positivity cut-offs or result categories of the index test, distinguishing pre-specified from exploratory	Page 6
	**12b**	Definition of and rationale for test positivity cut-offs or result categories of the reference standard, distinguishing pre-specified from exploratory	Page 6
	**13a**	Whether clinical information and reference standard results were available to the performers/readers of the index test	Page 6
	**13b**	Whether clinical information and index test results were available to the assessors of the reference standard	Page 6
Analysis	**14**	Methods for estimating or comparing measures of diagnostic accuracy	Page 7
	**15**	How indeterminate index test or reference standard results were handled	Page 7
	**16**	How missing data on the index test and reference standard were handled	Page 7
	**17**	Any analyses of variability in diagnostic accuracy, distinguishing pre-specified from exploratory	N/A

	**18**	Intended sample size and how it was determined	Page 4
**RESULTS**			
Participants	**19**	Flow of participants, using a diagram	Page 11
	**20**	Baseline demographic and clinical characteristics of participants	Page 9
	**21a**	Distribution of severity of disease in those with the target condition	Page 8
	**21b**	Distribution of alternative diagnoses in those without the target condition	Page 9
	**22**	Time interval and any clinical interventions between index test and reference standard	Page 10
Test results	**23**	Cross tabulation of the index test results (or their distribution) by the results of the reference standard	Page 12
	**24**	Estimates of diagnostic accuracy and their precision (such as 95% confidence intervals)	Page 11
	**25**	Any adverse events from performing the index test or the reference standard	Not applicable
**DISCUSSION**			
	**26**	Study limitations, including sources of potential bias, statistical uncertainty, and generalisability	Page 14
	**27**	Implications for practice, including the intended use and clinical role of the index test	Page 14
**OTHER INFORMATION**			
	**28**	Registration number and name of registry	Page 7
	**29**	Where the full study protocol can be accessed	Pols data policy
	**30**	Sources of funding and other support; role of funders	Author form

Comments to the Author

Reviewer #1:

The authors submitted a revised manuscript and responded to the questions. The authors stated that “The Alvarado score (AS) was not documented. However, based on symptoms assessed and the results of the CBC, the AS was computed for each patient. The mean AS was 4.64 (95% CI 4.32-4.97), those with AS of 1 to 4 were 50.6%”. As the authors’ replied, the inclusion criteria of the study were too loose. The AS of the patients less than 4 were 50.6% of the study patients, who should not receive abdominal CT for suspect acute appendicitis. The loose criteria made the high percentage (39%) of urolithiasis in the study population. The conclusion: “The commonest alternative diagnosis was urolithiasis and should be considered during the evaluation of adult patients with suspected acute appendicitis.” is inappropriate. Emergency physicians usually perform more examinations, such as urinalysis and abdominal ultrasonography for patients with right lower abdominal colic pain before CT. The diagnosis of urolithiasis is easy to identify by these examinations. If we adopt the authors’ criteria, we would perform CT for every patient with right lower abdominal colic pain.

Response:

Thank you for your comments. For uniformity, the inclusion criteria were adopted from a meta-analysis and systematic on the same subject by Bo Rud et al, 2019. The use of USS prior to CT introduces a confounding effect on the assessment of CT’s diagnostic performance, and this is an exclusion criterion, in this study and the systematic review quoted above.

The concerns on the use of AS have been analyzed and discussed, using the AS threshold of 4 would have resulted in 50% less CT scans, and missed 4 AA. Recommendations for the use of AS in assessing patients with suspected acute appendicitis have been emphasized.

The recommendation by the authors referred to above had been rectified in an earlier revised version and is not present in the current revised version. Results of urinalysis done have been described and analyzed.

Reviewer #2:

This is my second review of this manuscript. The authors have replied and made revisions regarding previous reviewers' major concerns. Yet there remained more issues.

1. In my first comment, the authors stated that "The image interpreters were four consultant medical radiologists rotating through an on-call schedule. Please give the Kappa value between these four radiologists". And the authors replied as "Each radiologist interpreted the results independently, depending on when he/she was on call. This has been clarified in the manuscript". For this comment, my question was "whether the same image was interpreted by two or more radiologists or just one? If two or more radiologists have interpreted the same image, I would like to know how the degree of the consistent outcome through giving the kappa values? But if just one radiologist interpreted for one image, this may be lead to an inconsistent outcome and must have been addressed.

2. In my first comment, I have stated that "Please give the 95% confidence intervals for sensitivity, specificity, diagnostic accuracy, positive and negative predictive value", but I suggest the authors revise the terms of confidence intervals into more formula terms. For example, (95% Confidence Interval of 0.79 to 0.96) to (95% C.I.: 0.79-0.96). the same terms through this manuscript like this term also please address accordingly. Additionally, do not use the percentage and decimal point interchangeably, please unify.

Response to comment 1.

The concern for four independent radiologists evaluating the CT introduces inter-observer variability. This is has been discussed as a limitation of this study.

Response to comment 2.

This has been rectified. Thank you for your comments.
