# Supplementary material for: Diagnostic accuracy of computed tomography in adults with suspected acute appendicitis at the emergency department in a private tertiary hospital in Tanzania
Source: PLoS One. 2022 Oct 27;17(10):e0276720. doi: 10.1371/journal.pone.0276720 (PMC9612457; doi:10.1371/journal.pone.0276720)
Supplement: Attachment — Submitted filename: Response to Reviewers.docx [file pone.0276720.s002.docx]

RESPONSE TO REVIEWERS


[Note: HTML markup is below. Please do not edit.]

Reviewers' comments:

Reviewer's Responses to Questions

**Comments to the Author**

1. Is the manuscript technically sound, and do the data support the conclusions?

The manuscript must describe a technically sound piece of scientific research with data that supports the conclusions. Experiments must have been conducted rigorously, with appropriate controls, replication, and sample sizes. The conclusions must be drawn appropriately based on the data presented.

Reviewer #1: Partly

Reviewer #2: Yes

2. Has the statistical analysis been performed appropriately and rigorously?

Reviewer #1: No

Reviewer #2: No

3. Have the authors made all data underlying the findings in their manuscript fully available?

The [PLOS Data policy](https://emea01.safelinks.protection.outlook.com/?url=http%3A%2F%2Fwww.plosone.org%2Fstatic%2Fpolicies.action%23sharing&data=04%7C01%7C%7Ce6a1319d0a2c41e52e2008d91a30d2a5%7C84df9e7fe9f640afb435aaaaaaaaaaaa%7C1%7C0%7C637569619261367943%7CUnknown%7CTWFpbGZsb3d8eyJWIjoiMC4wLjAwMDAiLCJQIjoiV2luMzIiLCJBTiI6Ik1haWwiLCJXVCI6Mn0%3D%7C1000&sdata=htTHRehf8vcNPaXijr%2BYtmjt%2BQF8zM277GR05%2FW6lNw%3D&reserved=0) requires authors to make all data underlying the findings described in their manuscript fully available without restriction, with rare exception (please refer to the Data Availability Statement in the manuscript PDF file). The data should be provided as part of the manuscript or its supporting information, or deposited to a public repository. For example, in addition to summary statistics, the data points behind means, medians and variance measures should be available. If there are restrictions on publicly sharing data—e.g. participant privacy or use of data from a third party—those must be specified.

Reviewer #1: Yes

Reviewer #2: No

4. Is the manuscript presented in an intelligible fashion and written in standard English?

PLOS ONE does not copyedit accepted manuscripts, so the language in submitted articles must be clear, correct, and unambiguous. Any typographical or grammatical errors should be corrected at revision, so please note any specific errors here.

Reviewer #1: Yes

Reviewer #2: No

5. Review Comments to the Author

Please use the space provided to explain your answers to the questions above. You may also include additional comments for the author, including concerns about dual publication, research ethics, or publication ethics. (Please upload your review as an attachment if it exceeds 20,000 characters)

Reviewer #1: The authors aimed to study the diagnostic accuracy of acute appendicitis (AA) by computed tomography (CT) in Tanzania. They recruited 180 patients suspected AA with CT retrospectively in a single hospital. The reference standards were histological examination of the removed appendix as well as clinical follow‐up for 14 days of participants who did not have surgery. After excluded 7 patients with negative CT and loss of follow-up, 173 participants were included in analysis of which 45 had and 128 did not have AA. The CT's diagnostic accuracy for AA was 97.5%, sensitivity and specificity were 91.6% and 100% respectively. The authors concluded the diagnostic accuracy was comparable to the studies conducted outside of Africa.

Comments:

1. The accuracy of CT to diagnose AA has been well documented. The authors hypothesized the equipment or experience in interpretation of CT in Tanzania, Africa might be different from the outside of Africa. The images of the study were generated by Philips, Ingenuity model, 128 slices with 64 detector rows. All 4 radiologists interpreting the images had at least trained as Master of Medicine in medical radiology. The equipment and experience in interpretation of CT in this hospital (Aga Khan Hospital) of Tanzania were at the same level as previous studies conducted outside of Africa. The results of this study are expected.

**There were no prior published studies examining the diagnostic accuracy of CT for acute appendicitis in the sub-Sahara Africa. Given the justification of this study that the assumption of similar performance might not necessarily be true, this question was explored.**

2. This is a retrospective study without clear inclusion criteria. The authors recruited the patients with right lower abdominal pain with suspected AA by physicians (no criteria about how to suspect AA) and underwent CT scans. The inaccurate diagnostic criteria before CT resulted in a high portion of patients without AA in the study. There were 45 case (26%) of AA and 68 cases (39%) of urolithiasis in the study population. The sample size estimation on page 12 used the prevalence of 0.43, which is much higher than 26% in the study population. Therefore, the sample size estimation was invalid.

**Criteria for suspecting acute appendicitis, right iliac fossa colicky abdominal pain of less than 10 days duration. This has been clarified in the manuscript.**

**As a result of the lower prevalence of acute appendicitis in this study, the corresponding precision is lowered to 0.067 as opposed to our sample size estimation of 0.05. This information has been added in the manuscript as a limitation of the study.**

3. The Fig. 1 illustrated the study population. At top of the Fig. 1, it displayed 7 cases of missing data were excluded. The rest of the Fig. 1 demonstrated the outcomes of the 180 cases. However, there were 7 cases with negative CT and loss of follow-up should be excluded from this study cohort. This exclusion was not demonstrated in the Fig. 1.

**Revised.**

Reviewer #2: This is a retrospective diagnostic accuracy study conducted at a private tertiary hospital in Tanzania. The authors aimed to assess the diagnostic accuracy of a computed tomography scan for acute appendicitis in patients with suspected acute appendicitis at the emergency department. They found that the high diagnostic accuracy (97.5%) was comparable to that demonstrated in meta-analysis from studies conducted outside of Africa. This reviewer is an applied statistician and methodologist. I have several concerns.

The entire manuscript needs to be closely edited by someone more proficient with the nuances of the English language as there are multiple sites in which the grammar could be improved.

1. Methodology- The period of this study conducted in the “Methodology” section was different from that recorded in “Abstract”. Which one was correct? Please revise.

**Revised.**

2. Methodology- The authors wrote that “Adult patients above ten years of age were included in the study….”. This is different from definition of adult (>14 years of age) in the SRMA conducted by Rud B, et al. (2019). Please explain more.

**In the setting in which the study was conducted, patients above the age of 10 years were managed with adult surgeons. On further review, there were a total of 4 participants below the age of 14, making this 2.2% of the sample size. This information has been added in the manuscript.**

3. Methodology- Presenting results for diagnostic test assessment include sensitivity, specificity, diagnostic odds ratio, and AUC. The authors should consider adding.

**Considerations given; ROC analysis not deemed necessary as the index test results and gold standard were either positive or negative (dichotomous) and not a scale.**

4. Methodology-The authors wrote that “The image interpreters were four consultant medical radiologists rotating through an on-call schedule”. Please give the Kappa value between these four radiologists.

**Each radiologist interpreted the results independently, depending on when he/she was on call. This has been clarified in the manuscript.**

5. Methodology- The authors wrote that “Sample size estimation based on Buderer formula of diagnostic accuracy for unknown disease prevalence”. But this is a retrospective study, why do this calculate? Please explain more.

**Sample size calculation was done to ascertain the confidence of the results and to know the sample size required to reach this confidence.**

6. Results-Please give a more detailed demographic table about these 180 included participants.

**Additional information provided.**

7. Results- The authors wrote that “the mean participants’ age was 35 years with a range (10 to 71)…”. Formally, for continuous variable like age, it must report as mean with standard deviation or 95% confidence interval. Please revise.

**Revised.**

8. Results- Please revise the form for reporting the proportion. As example, line 81 states that “the first assessor was a medical officer in 40 %, but line 83 states that “six percent of the …..”. It must be consistent.

**Revised.**

9. Results- Line 96-97 states that “A total of 35 participants determined to have normal appendices, without alternative diagnoses (27) and equivocal CT results (8) ….”. I think that the notations (27) and (8) stood for the numbers of participants but they were same as notations for cite references. Please revise.

**Revised.**

10. Results- Please unify the number part, don’t use English and Arabic numerals interchangeably.

**Revised.**

11. Results- In Line 108-111, Please give the 95% confidence intervals for sensitivity, specificity, diagnostic accuracy, positive and negative predictive value.

**Revised.**

12. Results- The authors wrote that “No significant differences were observed…”. Does any statistical method was used? Which value of p-value was considered to be statistically significant?

**This subgroup analysis has been omitted due to limited samples**

13. Can’t find the list of abbreviations.

**List of Abbreviations Added.**

6. PLOS authors have the option to publish the peer review history of their article ([what does this mean?](https://emea01.safelinks.protection.outlook.com/?url=https%3A%2F%2Fjournals.plos.org%2Fplosone%2Fs%2Feditorial-and-peer-review-process%23loc-peer-review-history&data=04%7C01%7C%7Ce6a1319d0a2c41e52e2008d91a30d2a5%7C84df9e7fe9f640afb435aaaaaaaaaaaa%7C1%7C0%7C637569619261377931%7CUnknown%7CTWFpbGZsb3d8eyJWIjoiMC4wLjAwMDAiLCJQIjoiV2luMzIiLCJBTiI6Ik1haWwiLCJXVCI6Mn0%3D%7C1000&sdata=f6%2BFAR2WXCdGaGI5M1RyeErSzEsrKoyLBGE13Z1ZUiw%3D&reserved=0)). If published, this will include your full peer review and any attached files.


If you choose “no”, your identity will remain anonymous but your review may still be made public.


**Do you want your identity to be public for this peer review?** For information about this choice, including consent withdrawal, please see our [Privacy Policy](https://emea01.safelinks.protection.outlook.com/?url=https%3A%2F%2Fwww.plos.org%2Fprivacy-policy&data=04%7C01%7C%7Ce6a1319d0a2c41e52e2008d91a30d2a5%7C84df9e7fe9f640afb435aaaaaaaaaaaa%7C1%7C0%7C637569619261377931%7CUnknown%7CTWFpbGZsb3d8eyJWIjoiMC4wLjAwMDAiLCJQIjoiV2luMzIiLCJBTiI6Ik1haWwiLCJXVCI6Mn0%3D%7C1000&sdata=qExSbAX78XD9yBBW%2FCXlYKs5TuqRSIfsKob1Hjb21D0%3D&reserved=0).

Reviewer #1: **Yes:** Cheng-Chung Fang

Reviewer #2: No

[NOTE: If reviewer comments were submitted as an attachment file, they will be attached to this email and accessible via the submission site. Please log into your account, locate the manuscript record, and check for the action link "View Attachments". If this link does not appear, there are no attachment files.]

While revising your submission, please upload your figure files to the Preflight Analysis and Conversion Engine (PACE) digital diagnostic tool, [https://pacev2.apexcovantage.com/](https://emea01.safelinks.protection.outlook.com/?url=https%3A%2F%2Fpacev2.apexcovantage.com%2F&data=04%7C01%7C%7Ce6a1319d0a2c41e52e2008d91a30d2a5%7C84df9e7fe9f640afb435aaaaaaaaaaaa%7C1%7C0%7C637569619261387927%7CUnknown%7CTWFpbGZsb3d8eyJWIjoiMC4wLjAwMDAiLCJQIjoiV2luMzIiLCJBTiI6Ik1haWwiLCJXVCI6Mn0%3D%7C1000&sdata=8gBvjZ4%2B8rChxzh38gOQia623br0kA5Tl6LJC3Op2E4%3D&reserved=0). PACE helps ensure that figures meet PLOS requirements. To use PACE, you must first register as a user. Registration is free. Then, login and navigate to the UPLOAD tab, where you will find detailed instructions on how to use the tool. If you encounter any issues or have any questions when using PACE, please email PLOS at [figures@plos.org](mailto:figures@plos.org). Please note that Supporting Information files do not need this step.

*In compliance with data protection regulations, you may request that we remove your personal registration details at any time.* [*(Remove my information/details)*](https://emea01.safelinks.protection.outlook.com/?url=https%3A%2F%2Fwww.editorialmanager.com%2Fpone%2Flogin.asp%3Fa%3Dr&data=04%7C01%7C%7Ce6a1319d0a2c41e52e2008d91a30d2a5%7C84df9e7fe9f640afb435aaaaaaaaaaaa%7C1%7C0%7C637569619261387927%7CUnknown%7CTWFpbGZsb3d8eyJWIjoiMC4wLjAwMDAiLCJQIjoiV2luMzIiLCJBTiI6Ik1haWwiLCJXVCI6Mn0%3D%7C1000&sdata=Fr9HGiPcn9ECTQRB38E9xHONRlv0HoKOBf8i1qtycbM%3D&reserved=0)*. Please contact the publication office if you have any questions.*
