# Supplementary material for: Diagnostic accuracy of computed tomography in adults with suspected acute appendicitis at the emergency department in a private tertiary hospital in Tanzania
Source: PLoS One. 2022 Oct 27;17(10):e0276720. doi: 10.1371/journal.pone.0276720 (PMC9612457; doi:10.1371/journal.pone.0276720)
Supplement: Attachment — Submitted filename: Response to Reviewers.docx [file pone.0276720.s003.docx]

**Response to Reviewer #1:** 
1. Details of evaluations performed by emergency medicine physicians including additional tests have been included in the manuscript.
2. The rationale has been refined further.

**Responses to Reviewer #2:** 
1. Grammatical and typographical errors reviewed and rectified.
2. To conform with the SMRA study, patients below the age of 14 have been excluded from the analysis of this study.
3. AUROC added and additional subgroup analyses.
4. The images were interpreted by one of four radiologists who was on call. This information has been clarified in the manuscript.
5. More data is certainly better. However, this also means more use of resources for data collection procedures, and sometimes this has ethical implications, hence calculating what would be needed allows one to know how much data collection is adequate. The lower prevalence of acute appendicitis in our study affected the precision of the results. This has been discussed in the discussion section.
6. Table one has been reviewed to contain more demographic details.
7. The reporting of results has been revised.
9. This section has been revised.
10. This section has been revised.
