# Supplementary material for: Diagnostic accuracy of computed tomography in adults with suspected acute appendicitis at the emergency department in a private tertiary hospital in Tanzania
Source: PLoS One. 2022 Oct 27;17(10):e0276720. doi: 10.1371/journal.pone.0276720 (PMC9612457; doi:10.1371/journal.pone.0276720)
Supplement: Attachment — Submitted filename: Response to reviewers.docx [file pone.0276720.s004.docx]

**Comments to the Author**

Reviewer #1:

The authors submitted a revised manuscript and responded to the questions. The authors stated that “The Alvarado score (AS) was not documented. However, based on symptoms assessed and the results of the CBC, the AS was computed for each patient. The mean AS was 4.64 (95% CI 4.32-4.97), those with AS of 1 to 4 were 50.6%”. As the authors’ replied, the inclusion criteria of the study were too loose. The AS of the patients less than 4 were 50.6% of the study patients, who should not receive abdominal CT for suspect acute appendicitis. The loose criteria made the high percentage (39%) of urolithiasis in the study population. The conclusion: “The commonest alternative diagnosis was urolithiasis and should be considered during the evaluation of adult patients with suspected acute appendicitis.” is inappropriate. Emergency physicians usually perform more examinations, such as urinalysis and abdominal ultrasonography for patients with right lower abdominal colic pain before CT. The diagnosis of urolithiasis is easy to identify by these examinations. If we adopt the authors’ criteria, we would perform CT for every patient with right lower abdominal colic pain.

Response:

Thank you for your comments. For uniformity, the inclusion criteria were adopted from a meta-analysis and systematic on the same subject by Bo Rud et al, 2019. The use of USS prior to CT introduces a confounding effect on the assessment of CT’s diagnostic performance, and this is an exclusion criterion, in this study and the systematic review quoted above.

The concerns on the use of AS have been analyzed and discussed, using the AS threshold of 4 would have resulted in 50% less CT scans, and missed 4 AA. Recommendations for the use of AS in assessing patients with suspected acute appendicitis have been emphasized.

The recommendation by the authors referred to above had been rectified in an earlier revised version and is not present in the current revised version. Results of urinalysis done have been described and analyzed.

Reviewer #2:

This is my second review of this manuscript. The authors have replied and made revisions regarding previous reviewers’ major concerns. Yet there remained more issues.
1. In my first comment, the authors stated that “The image interpreters were four consultant medical radiologists rotating through an on-call schedule. Please give the Kappa value between these four radiologists”. And the authors replied as “Each radiologist interpreted the results independently, depending on when he/she was on call. This has been clarified in the manuscript”. For this comment, my question was “whether the same image was interpreted by two or more radiologists or just one? If two or more radiologists have interpreted the same image, I would like to know how the degree of the consistent outcome through giving the kappa values? But if just one radiologist interpreted for one image, this may be lead to an inconsistent outcome and must have been addressed.
2. In my first comment, I have stated that “Please give the 95% confidence intervals for sensitivity, specificity, diagnostic accuracy, positive and negative predictive value”, but I suggest the authors revise the terms of confidence intervals into more formula terms. For example, (95% Confidence Interval of 0.79 to 0.96) to (95% C.I.: 0.79-0.96). the same terms through this manuscript like this term also please address accordingly. Additionally, do not use the percentage and decimal point interchangeably, please unify.

Response to comment 1.

The concern for four independent radiologists evaluating the CT introduces inter-observer variability. This is has been discussed as a limitation of this study.

Response to comment 2.

This has been rectified. Thank you for your comments.
