# Supplementary material for: Diagnostic accuracy of computed tomography in adults with suspected acute appendicitis at the emergency department in a private tertiary hospital in Tanzania
Source: PLoS One. 2022 Oct 27;17(10):e0276720. doi: 10.1371/journal.pone.0276720 (PMC9612457; doi:10.1371/journal.pone.0276720)
Supplement: Attachment — Submitted filename: Response to Reviewers.docx [file pone.0276720.s006.docx]

**Response to Reviewers**

**Reviewer #1:** The authors submitted the third revision of their manuscript. Same as the previous revisions, the authors did not directly respond to my questions, and they did not make point-to-point responses. The study population did not change after these revisions. Therefore, my questions were not resolved after the revisions. This study is a retrospective chart review study and has many inclusion biases when calculating the diagnostic accuracy of CT in acute appendicitis. I do not wish to make comments anymore, as my previous comments are still unresolved.

**Response to reviewer 1:** Thank you for your review. The risk of inclusion bias due to the retrospective nature of the study has been added as a limitation.

Below are the comments that were made on the previous review, and I have tried to break them down so that I may be able to provide appropriate responses

Reviewer #1:

The authors submitted a revised manuscript and responded to the questions. The authors stated that “The Alvarado score (AS) was not documented. However, based on symptoms assessed and the results of the CBC, the AS was computed for each patient.

1. The mean AS was 4.64 (95% CI 4.32-4.97), those with AS of 1 to 4 were 50.6%”. As the authors’ replied, the inclusion criteria of the study were too loose. The AS of the patients less than 4 were 50.6% of the study patients, who should not receive abdominal CT for suspect acute appendicitis. The loose criteria made the high percentage (39%) of urolithiasis in the study population.
2. The conclusion: “The commonest alternative diagnosis was urolithiasis and should be considered during the evaluation of adult patients with suspected acute appendicitis.” is inappropriate.
3. Emergency physicians usually perform more examinations, such as urinalysis and abdominal ultrasonography for patients with right lower abdominal colic pain before CT. The diagnosis of urolithiasis is easy to identify by these examinations.
4. If we adopt the authors’ criteria, we would perform CT for every patient with right lower abdominal colic pain.

**Response to Reviewer 1**:

1. For uniformity, the inclusion criteria were adopted from a meta-analysis and systematic on the same subject by Bo Rud et al, 2019, whereby the Alvarado Score was not used for participant selection. However, as noted Alvarado Score was not performed in routine clinical practice to evaluate patients with suspected Acute Appendicitis; it was calculated in this study based on the workup performed.

Had the AS been conducted in routine clinical practice and an AS of < 4 chosen to determine the need to perform CT, 50% fewer CT scans would have been conducted and missed 4 Acute Appendicitis. In the discussion, based on these results, we emphasise and advocate the routine use of AS to guide further management of patients with suspected AA.

2. This conclusion was removed in an earlier version and is not present in the current version.
3. Patients who underwent USS before CT were excluded from this study as this would introduce a confounding effect on CT’s diagnostic performance. Urinalysis was done in 134 patients and was normal in 79.1%, 19.4% showing microscopic hematuria, and pyuria with leucocytes in 1.5%. Urinalysis was not associated with a diagnosis of AA on binomial logistic regression (Positive for Acute Appendicitis or negative for Acute Appendicitis). This information is included in the revised manuscript
4. In the discussion section, we have emphasised and advocated the need to conduct an AS for patients with suspected AA before further management, as the results of this study also show that it would have decreased unnecessary CT scans.

**Reviewer #2**: This is my third review of this manuscript. The authors have replied and made many revisions regarding previous reviewers’ major concerns. I have no more comments. Thanks.

**Response to reviewer 2:** Thank you for your review.

**Reviewer #3:** Page 12, line 128: In the discussion section, the statement of "10239 hours" is incorrect, and should be "10239 minutes".

**Response to reviewer 3:** Thank you for the review. The change has been made

**Reviewer #4:** Dear authors, please see my comments in the attached pdf-file. You revised the manuscript several times before so I have focused on only important questions. I wish you the very best. Good Luck!

**Response to reviewer 4:** Thank you for the comments.

1. It is not unusual for patients to present late in this setting present due to various health-seeking behaviours, and we wanted to ensure we do not exclude them from the study due to health-seeking behaviour. However, by day 5, the cumulative percentage of patients who had presented to the EMD was 93%.
2. A medical officer is a registered medical doctor with a Doctor of Medicine degree qualification only. This definition has been added to the revised manuscript
3. A general surgery specialist was the first assessor in 23% of the cases. This has been included in the revised manuscript
4. We have rectified the noted errors in the placement of less than and greater than symbols.
5. Duration of illness was defined as the number of days from onset of abdominal pain to emergency medicine department orientation. The definition has been included in the revision.
